# Chinese herbal medicine for threatened miscarriage: An updated systematic review and meta-analysis

**DOI:** 10.3389/fphar.2023.1083746

**Published:** 2023-02-14

**Authors:** Hongliang Xie, Aolin Zhang, Xuan Mou, Tao He, Junwei Li, Chi Chiu Wang, Xiaohui Fan, Lu Li

**Affiliations:** ^1^ Pharmaceutical Informatics Institute, College of Pharmaceutical Sciences, Zhejiang University, Hangzhou, China; ^2^ Innovation Center in Zhejiang University, State Key Laboratory of Component-Based Chinese Medicine, Hangzhou, China; ^3^ Department of Obstetrics and Gynaecology, Li Ka Shing Institute of Health Sciences, School of Biomedical Sciences, Sichuan University-Chinese University of Hong Kong Joint Reproductive Medicine Laboratory, The Chinese University of Hong Kong, Shatin, China; ^4^ College of Basic Medical Sciences, Zhejiang Chinese Medical University, Hangzhou, China; ^5^ Future Health Laboratory, Innovation Center of Yangtze River Delta, Zhejiang University, Jiaxing, China; ^6^ Department of Obstetrics and Gynecology, Sir Run Run Shaw Hospital, Zhejiang University School of Medicine, Key Laboratory of Reproductive Dysfunction Management of Zhejiang Province, Hangzhou, China; ^7^ Institute of Chinese Medicine, The Chinese University of Hong Kong, Shatin, China

**Keywords:** Chinese herbal medicine, threatened miscarriage, systematic review, meta-analysis, efficacy, safety

## Abstract

**Objective:** To conduct an updated systematic review and meta-analysis on the efficacy and safety of Chinese herbal medicine (CHM) for threatened miscarriage.

**Data Sources:** Electronic databases were searched from inception to 30 June 2022. Study Eligibility Criteria: Only randomized controlled trials (RCTs) that assessed the efficacy and safety of CHM or combined CHM and Western medicine (CHM-WM) and compared with other treatments for threatened miscarriage were included for analysis.

**Methods:** Three review authors independently evaluated included studies, assessed the risk of bias and extracted data for meta-analysis (continuation of pregnancy after 28 gestational weeks, continuation of pregnancy after treatment, preterm birth, adverse maternal outcomes, neonatal death, TCM syndrome severity, β-hCG levels after treatment), sensitivity analysis (β-hCG level) and subgroup analysis (TCM syndrome severity, β-hCG level). The risk ratio and 95% confidence interval were calculated by RevMan. Certainty of the evidence was assessed according to GRADE.

**Results:** Overall, 57 RCTs involving 5,881 patients met the inclusion criteria. Compared with WM alone, CHM alone showed significant higher incidence of continuation of pregnancy after 28 gestational weeks (Risk Ratio (RR) 1.11; 95% CI 1.02 to 1.21; n = 1; moderate quality of evidence), continuation of pregnancy after treatment (RR 1.30; 95% CI 1.21 to 1.38; n = 10; moderate quality of evidence), higher β-hCG level (Standardized Mean Difference (SMD) 6.88; 95% CI 1.74 to 12.03; n = 4) and lower Traditional Chinese medicine (TCM) syndrome severity (SMD −2.94; 95% CI −4.27 to −1.61; n = 2). Compared with WM alone, combined CHM-WM showed significant higher incidence of continuation of pregnancy after 28 gestational weeks (RR 1.21; 95% CI 1.16 to 1.27; n = 15; moderate quality of evidence), continuation of pregnancy after treatment (RR 1.19; 95% CI 1.16 to 1.23; n = 41; moderate quality of evidence), higher β-hCG level (SMD 2.27; 95% CI 1.72 to 2.83; n = 37) and lower TCM syndrome severity (SMD −1.74; 95% CI −2.21 to −1.27; n = 15). No significant differences in reducing the adverse maternal outcomes and neonatal death were found in combined CHM-WM compared with WM alone (RR 0.97; 95% CI 0.62 to 1.52; n = 8; RR 0.39; 95% CI 0.12 to 1.21; n = 2).

**Conclusion:** Current evidence supported CHM could be a potential treatment for threatened miscarriage. However, results should be interpreted with caution considering the low to moderate quality of the available evidence.

**Systematic Review Registration:** [https://inplasy.com/inplasy-2022-6-0107/], identifier [INPLASY20220107].

## 1 Introduction

Threatened miscarriage is the most common pregnancy complication with an incidence of about 15%–20% and 20%–25% of the cases end up in spontaneous miscarriage. [(Wahabi et al., 2018), (Kouk et al., 2013)] Women with threatened miscarriages are 2.5 times more likely to miscarry than healthy women, which causes a huge physical and mental stress on women and their families. [(Sotiriadis et al., 2004), (Makrydimas et al., 2003)].

In clinical practice, many interventions have been used to prevent threatened miscarriage including bed rest (the most routinely prescribed intervention), anti-D immunoglobulin, endocrine regulation (e.g., the use of estrogen, progesterone, β-hCG), assisted reproductive technology, *etc.* [(Devall et al., 2021), (Sotiriadis et al., 2004), (Wahabi et al., 2018), (Aleman et al., 2005)] Although various interventions have been used to treat threatened miscarriage, most of them lack sufficient high-quality evidence to support their efficacy due to the unclear etiology and pathogenesis of threatened miscarriage. Thus, alternative medicines are greatly needed especially when Western medicine (WM) and other treatments are unable to provide a satisfactory therapeutic effect.

In China, Chinese herbal medicine (CHM) has been widely used in threatened miscarriage treatment for a long time. [(Giovanni, 1989)] For instance, “Shou Tai Wan (Quiet Foetus Pill)” or “An Tai Yin (Quiet Foetus Drink)” showed good protective effects against threatened miscarriage. (Chuang et al., 2007). Research showed that CHM can improve maternal-fetal immune ability, reduce the occurrence of inflammatory reactions and improve the level of endocrine in threatened miscarriage treatment. [(Ushiroyama et al., 2006), (Shen et al., 2022), (Deng et al., 2011)] Compared with WM alone, combined CHM-WM can achieve better curative effects for treating threatened miscarriage and no significant differences were found in adverse effects (Li et al., 2012a) However, more high-quality clinical evidence is needed to verify the effectiveness and safety of CHM on threatened miscarriage.

In our previous study, we made a systematic evaluation of the effectiveness of CHM for threatened miscarriage in 2012. (Li et al., 2012b). The meta-analysis showed a combination of CHM and WM was more effective than WM alone for treating threatened miscarriage, but all trials were methodologically poor and at unclear risk of bias overall. A large amount of randomized controlled trials (RCTs) was published over the past few years (Han et al., 2014; Duan et al., 2016; He, 2017; Guo et al., 2020; He, 2020; Cao and Yan, 2021; Dong and Zhang, 2021). Thus, we plan to update our review and systematically evaluate the effectiveness and safety of CHM for threatened miscarriage, ultimately providing more scientific and effective guidance for the clinical treatment of threatened miscarriage.

## 2 Methods

### 2.1 Study design

This systematic review and meta-analysis was conducted following a prospectively registered protocol (International Platform of Registered Systematic Review and Meta-analysis Protocols [INPLASY], INPLASY number INPLASY202260107, Supplementary Appendix A) and the Preferred Reporting Items for Systematic Reviews and Meta-Analyses (PRISMA) guidelines (PRISMA Checklist 2020; Supplementary Appendix B).

### 2.2 Eligibility criteria

#### 2.2.1 Types of studies

Only randomized controlled trials (RCTs) were included. Quasi‐randomized, cluster‐randomized trials, non-randomized trials, observational studies and cross‐over trials were excluded. There were no language restrictions among all the included RCTs.

#### 2.2.2 Types of patients

Women with threatened miscarriage at or before 28 gestational weeks regardless of underlying causes were included.

#### 2.2.3 Types of interventions

All types of CHM in either standard or combined regimens for the treatment of threatened miscarriage, regardless of the dose or duration of administration, were compared with placebo, no treatment or WM. We planned the following comparisons.

• CHM *versus* placebo.
• CHM *versus* no treatment (including bed rest).
• CHM alone *versus* WM alone.
• Combined CHM and WM (CHM-WM) *versus* WM alone.

#### 2.2.4 Types of outcome measures

Primary outcome

##### 2.2.4.1 Continuation of pregnancy after 28 gestational weeks

Pregnancy after 28 gestational weeks is generally considered viable, and miscarriage before 28 weeks is considered non-viable due to the extremely low birth weight and underdevelopment. In this review, only viable pregnancies and continuation of pregnancy after 28 gestational weeks were considered as the primary outcome.

The incidence of continuation of pregnancy after 28 gestational weeks = (total cases-cases of miscarriage)/total cases × 100%.

Secondary outcomes

##### 2.2.4.2 Continuation of pregnancy after treatment

The incidence of continuation of pregnancy after treatment = (total cases-ineffective cases)/total cases × 100%.

Ineffective cases: no significant decrease in abdominal pain, vaginal bleeding and other symptoms, but even aggravated. B ultrasound showed that the embryo size did not conform to the gestational age. (National Administation of Traditional Chinese Medidine, 2002).

##### 2.2.4.3 Preterm birth

The incidence of preterm birth = preterm birth cases/total cases × 100%.

##### 2.2.4.4 Adverse maternal outcomes

Patients were reported to have nausea, vomiting, headaches, mouth dryness, constipation, insomnia, diarrhea, rash, breast swelling and pain, *etc.* after treatment.

##### 2.2.4.5 Adverse neonatal outcomes

Neonatal were reported to have malformation, jaundice and genital bleeding, *etc.* after treatment.

##### 2.2.4.6 Neonatal death

The incidence of neonatal death = neonatal death cases/total cases × 100%.

##### 2.2.4.7 TCM syndrome severity

TCM syndrome severity was evaluated by TCM syndrome score. (National Administation of Traditional Chinese Medidine, 2002). The TCM syndrome score was recorded and graded based on the degree of individual symptoms and all indicators were determined by comparing the values obtained after treatment with the baseline values. The scores of TCM syndromes were recorded according to the standards in ‘Guiding Principles for Clinical Research of New Chinese Medicine’. (National Administation of Traditional Chinese Medidine, 2002). According to the severity of symptoms, the scores were calculated from a 0–3 scale: 0 as light, 1 as mild, 2 as moderate and 3 as severe. Higher scores indicated severer symptoms. (Zheng, 2002).

##### 2.2.4.8 Improvement in beta-human chorionic gonadotropin (β-hCG) levels

Research reported that the well-developed embryos in early pregnancy indicated β-hCG doubly increasing every other day. [(Brady et al., 2020), (Zhong et al., 2000), (Batzer et al., 1983), (Barnhart et al., 2004)] The level of β-hCG varies from individual to individual. With the prolongation of pregnancy, β-hCG will continue increasing, generally reaching a peak around the 10th week of pregnancy, and then decreasing. A significant increase in β-hCG predicts a successful pregnancy. While an abnormally low β-hCG level before 24 gestational weeks was associated with a risk of spontaneous loss. [(Braunstein et al., 1978), (Dugoff et al., 2004)]

### 2.3 Information sources, and search strategy

Databases including PubMed, Cochrane Pregnancy and Childbirth’s Trials Register, VIP, Central, Embase, Medline, China National Knowledge Infrastructure (CNKI), and WanFang Database were searched for all published RCTs. The search for the previous publication ended on 31 January 2012. Therefore, we searched from 1 February 2012 until 30 June 2022. Search strategies were designed with terms related to “threatened miscarriage”, “CHM”, “WM”, “RCT”, *etc.* No language restrictions were used. For the complete search strategy, see Supplementary Appendix C.

### 2.4 Study selection

**FIGURE 1:**
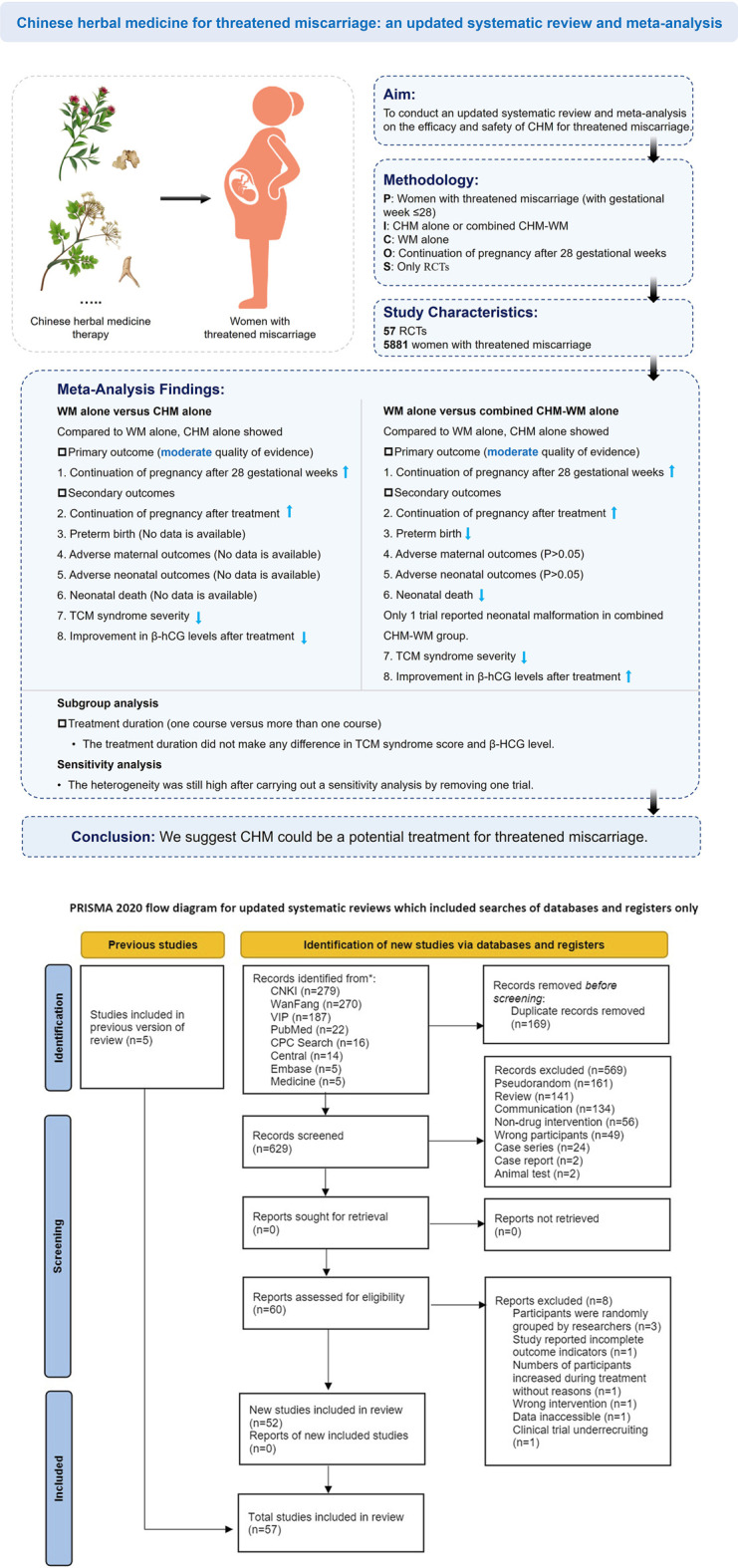
Flow diagram of study selection.

Two review authors (HL. Xie and AL. Zhang) independently assessed each trial for inclusion and any disagreements were resolved through discussion. If the disagreements could not be resolved, the arbiter (L. Li) made a final decision on the selected study. Details of the study selection were shown in the PRISMA study flow diagram, see Figure 1.

### 2.5 Data extraction

Two review authors (HL. Xie and AL. Zhang) developed a checklist for data recording, and independently extracted data through a standardized eligibility form. We resolved discrepancies through discussion or consulted the arbiter (L. Li) if necessary. When the data of RCTs were insufficient or ambiguous, we contacted corresponding authors for detailed information.

### 2.6 Risk of bias assessment

Two review authors (HL. Xie and AL. Zhang) independently performed the risk of bias using the Risk of Bias 2 (RoB-2) tool. (Sterne et al., 2019). It included five domains: bias arising from the randomization process, bias due to deviations from intended interventions, bias due to missing outcome data, bias in measurement of the outcome and bias in selection of the reported result. Each domain was assessed at low, or high risk of bias, or can be expressed as “some concerns”.

### 2.7 Data synthesis

We used Review Manager software (RevMan 5.4.1, 2020) for statistical analysis. Risk ratio (RR) and 95% confidence interval (CI) were used to analyze the effect size of dichotomous data. Besides, we used the standardized mean difference (SMD) to eliminate inconsistencies in units of measurement and measurement variances. I^2^ quantitative tests were used to test the heterogeneity among the RCTs. When *p* < 0.10, I^2^ > 50%, it suggested that there was high heterogeneity between studies, and the random-effect model shall be selected for meta-analysis. When *p* > 0.10, I^2^ < 50%, no obvious heterogeneity is suggested, and the fixed-effect model was selected for meta-analysis. Subgroup analysis was performed on the treatment course (short-term treatment (one course only) *versus* long-term treatment (more than one course)); gestational weeks (≤12 *versus*>12). As a sensitivity analysis, estimates were performed by excluding studies and analyzing the remaining studies to test the robustness of our results. The certainty of outcomes was interpreted using the Grading of Recommendations, Assessment, Development and Evaluations (GRADE) approach. [(GRADEpro, 2021), (The GRADE working group Schünemann et al., 2013)].

## 3 Results

### 3.1 Study selection

A total of 798 clinical studies were identified by our updated search. After the removal of duplicates, 629 studies were screened by title and/or abstract, and 569 studies were excluded initially according to the inclusion and exclusion criteria. Full texts of 60 studies were further reviewed, and 8 studies were further excluded with the reasons: 1 study only had abstract without accessible data; (Chen, 2013) 3 studies had the wrong randomization that the patients were randomly grouped by researchers;[(Xie, 2016), (Liu, 2015), (Yu and Yang, 2017)] 1 study reported incomplete outcome; (Zhang and Liu, 2016); 1 study increased the number of patients after randomization, thus we didn’t know how many patients were involved in different outcome assessment; (Tian and Chen, 2016); 1 study used TCM nursing which is inappropriate for our types of intervention; (Zhou and Jiang, 2015) 1 study was an ongoing study without accessible data. (Wu, 2021) We also re-assessed the 44 RCTs included and 2 RCTs awaiting classification in the previously published review. Only 5 RCTs meet the inclusion in this update, therefore, a total of 57 RCTs including 5,881 patients were included in this updated review, see Figure 1. Amongst them, 11 RCTs (involving 1,029 women) compared CHM alone with WM alone, and 46 RCTs (involving 4,852 women) compared combined CHM-WM with WM alone. [(Cao and Yan, 2021), (Dong and Zhang, 2021), (Duan et al., 2016), (Guo et al., 2020), (Han et al., 2014), (He, 2017), (He, 2020), (Huang, 2020), (Jiang, 2019), (Ju, 2017), (Kang and Wang, 2018), (Kong et al., 2021), (Lai et al., 2020a), (Lai et al., 2020b), (Li et al., 2004), (Li et al., 2006), (Li, 2019), (Li et al., 2020), (Liu et al., 2012), (Liu and Cui, 2016), (Liu, 2018), (Liu et al., 2020), (Liu, 2020), (Liu, 2021), (Long, 2018), (Luo, 2020), (Ma, 2019), (Mi et al., 2021), (Nong, 2019), (Shang et al., 2021), (Song and Ni, 2013), (Song et al., 2018), (Su, 2016), (Teng, 2018), (Wang and Niu, 2019), (Wu et al., 2017), (Xiao, 2008), (Xie, 2014), (Xin et al., 2018), (Yang, 2006), (Yang, 2012), (Ye and Tao, 2021), (Yu, 2018), (Yu et al., 2019), (Yu et al., 2021), (Yu and Jiang, 2021), (Xiao, 2008), (Zhang and Ding, 2015), (Zhang, 2017), (Zhang et al., 2019), (Zhang, 2020), (Zhang and Hou, 2020), (Zhang, 2021a), (Zhang, 2021b), (Zheng et al., 2020), (Zhu and Wang, 2019), (Zhuang, 2016)] We did not find any comparisons of CHM with placebo or no treatment in the included studies. WM treatments included tocolytic drugs (e.g., aspirin and dydrogesterone), hormonal supplementations (e.g., HCG and progesterone) and supportive supplements (e.g., vitamin E and folic acid). CHM treatments included Chinese formulae (e.g., Jiawei Shoutai pill, Zishen Yutai pill, Shoutai Yigong powder, Guben Antai decoction, *etc.*), as shown in Table 1 and Table 2. These Chinese formulae were composed of different botanical drugs, of which the detailed information was listed in Supplementary Appendix D.

**TABLE 1:** Study characteristics for included studies of CHM alone vs. WM alone.

Study ID	Randomization	Sample size		Gestational week	Intervention	Treatment duration	Outcomes (CHM alone vs. WM alone)							
		T	C		T	C		①	②	③	④	⑤	⑥	⑦
Duan et al. (2016)	Random number table	54	54	7–12	Bushen JianpiAntai decoction	Progesterone injection	14 days	NR	↑*	NR	NR	NR	↓*	NR
Jiang, (2019)	Random number draw	42	42	12–27	Chinese medicine formula	Progesterone + hCG injection	NR	NR	↑*	NR	NR	NR	NR	NR
Li et al. (2020)	Random number table	82	84	6–8	Jiawei Shoutai pill	Dydrogesterone tablets+ hCG injection	14 days	↑*	↑*	NR	NR	NR	NR	NR
Liu et al. (2012)	Random number table	30	30	6–8	Antai decoction	Progesterone + hCG injection	14 days	NR	↑*	NR	NR	NR	NR	NR
Luo, (2020)	Random number table	34	34	NR	Buqi Antaidecoction	Progesterone capsules	14 days	NR	↑*	NR	NR	NR	NR	↑*
Song and Ni, (2013)	Random number table	40	30	7–12	Shoutai pill andShaoyao Gancaodecoction	Progesterone + hCGinjection	20 days	NR	↑*	NR	NR	NR	NR	NR
Song et al. (2018)	Random number table	50	50	7–10	Yuetai decoction	Progesterone injection	10 days	NR	↑*	NR	NR	NR	↓*	↑*
Wu et al. (2017)	Random number table	60	60	6–8	Yunbao decoction	Yunkang oral liquid	14 days	NR	↑*	NR	NR	NR	NR	↑*
Xie, (2014)	Random number table	52	53	7–12	Anzi decoction	Progesterone injection	10 days	NR	↑*	NR	NR	NR	NR	NR
Yang, (2012)	Random number table	39	40	NR	Antai decoction	Progesterone capsules	14 days	NR	↑*	NR	NR	NR	NR	NR
Yu, (2018)	Random number table	35	34	6–10	Antai Fangloudecoction	Progesterone injection	15 days	NR	NR	NR	NR	NR	↓*	↑*

Abbreviations: T: treatment; C: control; hCG: human chorionic gonadotropin; ① continuation of pregnancy after 28 weeks of gestation; ② continuation of pregnancy after treatment; ③ preterm birth; ④ adverse maternal outcomes; ⑤ neonatal death; ⑥ TCM, syndrome severity; ⑦ β-hCG, levels; NR: not reported; ↑: increase; ↓: decrease; *: *p* < 0.05, **: *p* < 0.01 (*t*-test).

**TABLE 2:** Study characteristics for included studies of combined CHM-WM vs. WM alone.

Study ID	Randomization	Sample size		Gestational week	Intervention		Treatment duration	Outcomes (CHM alone vs. WM alone)						
		T	C		T	C		①	②	③	⑥	⑤	⑥	⑦
Cao and Yan, (2021)	Random number table	59	59	7–12	Jiawei Shoutai pills + C	Progesterone injection	20 days	↑*	↑*	↓*	NR	↓*	↓*	↑*
Dong and Zhang, (2021)	Random number table	50	50	NR	Zishen Yutai pills + C	Progesterone soft capsules	14 days	↑*	↑*	NR	*p* > 0.05	NR	NR	↑*
Guo et al. (2020)	Random number table	80	80	6–12	Xionggui Jiaoai decoction + C	Progesterone capsules	14 days	NR	↑*	NR	NR	NR	↓*	↑*
Han et al. (2014)	Random number table	70	70	NR	Gushen Antai pills + C	Progesterone capsules	14 days	NR	↑*	NR	NR	NR	NR	↑*
He, (2017)	Random number table	31	31	5–12	Shoutai Yigong powder + C	Progesterone capsules	10 days	NR	↑*	NR	NR	NR	↓*	↑*
He, (2020)	Random number table	70	70	6–9	Guben Antai decoction + C	Dydrogesterone tablets	10 days	↑*	NR	*p* > 0.05	NR	NR	NR	↑*
Huang, (2020)	Random number table	39	39	5–11	Gushen Antai pills + C	Dydrogesterone tablets + Progesterone injection	14 days	↑*	↑*	NR	NR	NR	NR	↑*
Ju, (2017)	Random number table	300	200	NR	Gushen Antai pills + C	Progesterone capsules	10 days	NR	↑*	NR	NR	NR	NR	↑*
Kang and Wang, (2018)	Random number table	60	60	3–10	Yangxue Antai decoction + C	Progesterone capsules	28 days	NR	↑*	NR	NR	NR	↓*	NR
Kong et al. (2021)	Random number table	40	40	5–10	Bushen Huoxue recipe + C	Dydrogesterone tablets	15 days	↑*	↑*	NR	NR	NR	↓*	
Lai et al. (2020a)	Random number table	41	41	<12	Chinese medicine formula + C	Progesterone injection	14 days	NR	↓*	NR	NR	NR	NR	↑*
Lai et al. (2020b)	Random number table	41	41	5–10	Shoutai pill + C	Progesterone injection	20 days	↑*	NR	NR	NR	NR	↓*	↑*
Li et al. (2004)	Stratified Random Method	25	25	5–8	Bushen Antai Yin + C	Progesterone injection	NR	NR	↑*	NR	NR	NR	NR	↑*
Li et al. (2006)	Random number table	45	44	5–10	Bushen Gutai decoction + C	Progesterone injection	10 days	NR	↑*	NR	NR	NR	NR	NR
Li, (2019)	Random number table	50	50	6–7	Chushi Antai recipe + C	Progesterone injection	14 days	↑*	↑*	↓*	NR	NR	↓*	↑*
Liu and Cui, (2016)	Random number table	33	32	6	Bushen Jianpi recipe + C	Progesterone injection	21 days	NR	↑*	NR	NR	NR	↓**	↑*
Liu, (2018)	Randomization software	42	42	6–9	Bushen Yiqi Zhitong Antai recipe + C	Progesterone injection	14 days	NR	↑*	NR	NR	NR	NR	↑*
Liu et al. (2020)	Random number table	40	40	6–12	Guben Antai decoction + C	Progesterone capsules	NR	NR	↑*	↓*	*p* > 0.05	NR	↓*	↑*
Liu, (2020)	Random number table	30	30	7–9	Antai pill + C	Diquprogesterone tablets		↑*	NR	NR	*p* > 0.05	NR	↓*	↑*
Liu, (2021)	Random number table	40	40	5–8	Zishen Yangtai recipe + C	Conventional treatment	14 days	↑*	NR	NR	NR	NR	↓*	↑*
Long, (2018)	Random number table	60	60	NR	Bushen Huoxue recipe + C	Progesterone injection	14 days	↑*	↑*	↓*	NR	NR	NR	↑*
Ma, (2019)	Random number table	64	64	7–28	Guben Antai decoction + C	Progesterone capsules	14 days	↑*	↑*	↓*	*p* > 0.05	NR	↓*	↑*
Mi et al. (2021)	Random number table	58	58	7–12	Guyuan Wentai decoction + C	Progesterone injection	14 days	NR	↑*	NR	NR	NR	NR	↑*
Nong, (2019)	Random number table	55	55	6–13	Chinese medicine formula + C	Progesterone injection	14 days	↑*	↑*	NR	NR	NR	↓*	NR
Shang et al. (2021)	Random number table	40	40	4–11	Gushen Antai decoction + C	Dydrogesterone tablets	14 days	↑*	↑*	NR	*p* > 0.05	NR	NR	↑*
Su, (2016)	Random number table	60	60	4–12	Bushen Gutai decoction + C	Progesterone injection	7 days	NR	↑*	NR	NR	NR	NR	↑*
Teng, (2018)	Random number table	60	60	5–18	Xuanyu Tongjing decoction + C	Progesterone injection	21 days	↑*	↑*	NR	*p* > 0.05	NR	NR	↑*
Wang and Niu, (2019)	Random number table	40	40	5–12	Bushen Antai decoction + C	Progesterone injection	7 days	NR	↑*	NR	NR	NR	NR	↑*
Xiao, (2008)	Random number table	30	30	0–12	BuqiYangxue Gushen decoction + C	Progesterone injection	7 days	NR	↑*	NR	NR	NR	NR	NR
Xin et al. (2018)	Envelope Method	60	60	NR	Zishen Yutai pills + C	hCG injection + Vitamin E	14 days	↑*	↑*	↓*	NR	NR	NR	↑*
Yang, (2006)	Random number table	100	50	6–12	Chinese medicine formula + C	hCG injection + Vitamin E	NR	NR	↑*	NR	NR	NR	NR	NR
Ye and Tao, (2021)	Random number table	73	73	<12	Yangxue Gutai decoction + C	Dydrogesterone tablets	14 days	↑*	↑*	↓*	NR	*p* > 0.05	↓*	↑*
Yu et al. (2019)	Random number table	59	59	7–9	Chinese medicine formula + C	Dexamethasone tablets	21 days	NR	↑*	NR	NR	NR	NR	↑*
Yu et al. (2021)	Random number table	50	50	NR	Zishen Yutai pills + C	Dydrogesterone tablets	21 days	NR	↑*	NR	NR	NR	↓*	↑*
Yu and Jiang, (2021)	Random number table	30	30	6–10	Bushen Antai granules + C	Dydrogesterone tablets	14 days	NR	↑*	NR	NR	NR	↓*	NR
Xiao, (2008	Random number table	50	46	0–12	Shoutai pills + C	hCG injection	7 days	NR	↑*	NR	NR	NR	NR	NR
Zhang and Ding, (2015	Random number table	50	50	6–9	Chinese medicine formula + C	Progesterone injection	21 days	NR	↑*	NR	NR	NR	↓*	↑*
Zhang, (2017)	Random number table	20	20	6–9	Wushan Yangshi Baotai decoction + C	Progesterone injection	21day	NR	↑*	NR	NR	NR	↓*	↑*
Zhang et al. (2019)	Random number table	60	60	6–16	Bushen Jianpi recipe + C	Progesterone injection	20 days	NR	↑*	NR	NR	NR	NR	NR
Zhang, (2020)	Random number table	42	42	NR	Yuyin decoction + C	Progesterone capsules	21 days	NR	↑*	NR	*p* > 0.05	NR	NR	↑*
Zhang and Hou, (2020)	Random number table	30	30	NR Zhang 2020a	Baoyin decoction + C	Dydrogesterone tablets	14 days	↑*	↑*	NR	NR	NR	NR	↑*
Zhang, (2021a)	Random number table	40	40	NR	Yuantu Gutai decotion + C	Progesterone injection	30 days	NR	NR	NR	NR	NR	↓*	↑*
Zhang, (2021b)	Random number table	42	42	NR	Shoutai pills + C	Dydrogesterone tablets	14 days	NR	↑*	NR	NR	NR	↓*	↑*
Zheng et al. (2020)	Random number table	40	40	6–10	Bushen Yiqi Guchong Antai decoction + C	Progesterone injection	14 days	NR	↑*	NR	NR	NR	↓*	↑*
Zhu and Wang, (2019)	Random number table	51	51	<12	Bushen Baotai Zhuyun decoction + C	Dydrogesterone tablets	14 days	↑*	↑*	↓*	*p* > 0.05	NR	↓*	↑*
Zhuang, (2016)	Random number table	54	54	8–11	Yishen An Gong recipe + C	Progesterone injection	21 days	NR	NR	NR	NR	NR	↓*	*p* > 0.05

Abbreviations: T: treatment; C: control; hCG: human chorionic gonadotropin; ① continuation of pregnancy after 28 weeks of gestation; ② continuation of pregnancy after treatment; ③ preterm birth; ④ adverse maternal outcomes; ⑤ neonatal death; ⑥ TCM, syndrome severity; ⑦ β-hCG, levels; NR: not reported; ↑: increase; ↓: decrease; *: *p* < 0.05, **: *p* < 0.01 (*t*-test).

### 3.2 Study characteristics

The included RCTs were conducted in different cities in China. Both inpatients and outpatients were included. Although all the included studies did not report the registration of a clinical trial, they all received ethical approval following Chinese Good Clinical Practice. (gongbao, 2020). No trials included in our analysis have been retracted. Three studies reported there was no conflict of interest, [(Lai et al., 2020b), (Shang et al., 2021), (Zheng et al., 2020)] while other studies did not report whether there was a conflict of interest. A summary of the characteristics of 57 RCTs including randomization, method, patients, interventions and outcomes was shown in Table 1 and Table 2.

### 3.3 Risk of bias of included studies

The results of the RoB-2 evaluation were presented in Supplementary Figures S1,S2. No study was judged to be at low risk of bias for all 5 domains. The most common shortcoming was the failure to report the allocation sequence concealment. It was impossible to carry out the blinding for CHM in the clinical trials because patients in China receiving CHM insist on knowing the specific drugs to ensure drug safety and the potential harm to their pregnancies. Besides, there are obvious differences between CHM and WM in packaging, way of administration and taste. However, because most outcomes were objectively measured, the included RCTs were considered at a ‘low’ risk of bias despite a lack of outcomes assessment blinding. We judged the domain of reporting at low risk of bias even though the pre-specified plan of each included trial is inaccessible or unavailable. Because according to Good Clinical Practice of China, clinical trials must provide a trial protocol including ethics approval and must be reviewed and approved by experts before they were carried out. [92] Overall, all RCTs were rated as having an unclear risk of bias in allocation concealment and judged to be at low risk of bias for the other four domains.

### 3.4 Synthesis of results

#### 3.4.1 CHM alone group *versus* WM alone group

Primary outcome

##### 3.4.1.1 Continuation of pregnancy after 28 gestational weeks

**FIGURE 2:**
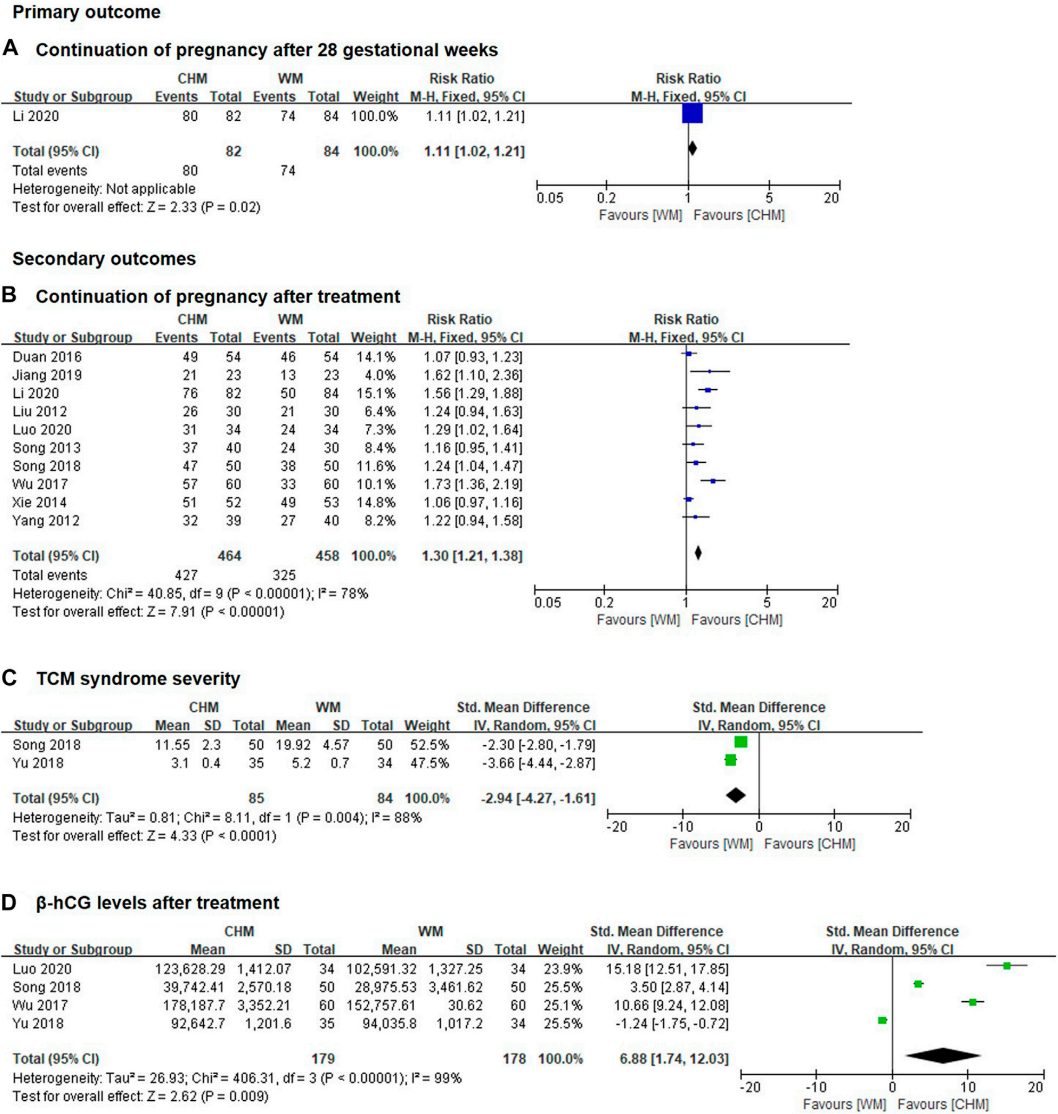
Forest plot of CHM alone group vs. WM alone group **(A)** continuation of pregnancy after 28 gestational weeks; **(B)** continuation of pregnancy after treatment; **(C)** TCM syndrome severity; **(D)** β-hcG levels after treatment.

Only 1 study including 166 patients reported the incidence of continuation of pregnancy after 28 gestational weeks was significantly increased in CHM alone group compared with WM alone group (RR 1.11, 95% CI 1.02 to 1.21; *p* = 0.02, Figure 2A). (Li et al., 2020) The certainty of the evidence was judged as moderate due to imprecision caused by few patients, see Supplementary Table S1.

Secondary outcomes

##### 3.4.1.2 Continuation of pregnancy after treatment

Ten trials including 922 patients reported the incidence of continuation of pregnancy after treatment was significantly increased in CHM alone group compared with WM alone group (RR 1.30, 95% CI 1.21 to 1.38, I^2^ = 78%, *p* < 0.00001, Figure 2B). (Liu et al., 2012; Yang, 2012; Song and Ni, 2013; Xie, 2014; Duan et al., 2016; Wu et al., 2017; Song et al., 2018; Jiang, 2019; Li et al., 2020; Luo, 2020). The certainty of the evidence was judged as moderate due to imprecision caused by few patients, see Supplementary Table S2.

##### 3.4.1.3 Preterm birth

No data is available for preterm birth in this comparison group.

##### 3.4.1.4 Adverse maternal outcomes

No data is available for adverse maternal outcomes in this comparison group.

##### 3.4.1.5 Adverse neonatal outcomes

No data is available for adverse neonatal outcomes in this comparison group.

##### 3.4.1.6 Neonatal death

No data is available for neonatal death in this comparison group.

##### 3.4.1.7 TCM syndrome severity

SMD with 95% CI was used as a measurement to eliminate the effect of dimension because different definitions of TCM syndrome severity were reported in different studies. Two trials included 169 patients reported the TCM syndrome severity was significantly reduced in CHM alone group compared with WM alone group (SMD -2.94, 95% CI -4.27 to −1.61, I^2^ = 88%, *p* < 0.0001, Figure 2C). (Song et al., 2018; Yu, 2018). The certainty of the evidence for this outcome was assessed as low due to imprecision caused by a wide 95% CI and a small number of trials and patients, see Supplementary Table S1.

##### 3.4.1.8 Improvement in β-hCG levels after treatment

SMD with 95% CI was used as a measurement to eliminate the effect of dimension because the data of β-hCG extracted from different studies were reported using different units. Research has shown that a higher level of β-hCG was associated with a better pregnancy success rate. Four clinical trials including 357 patients reported that the level of β-hCG was significantly increased in CHM alone group compared with WM alone group after treatment (SMD 6.88, 95% CI 1.74 to 12.03, I^2^ = 99%, *p* = 0.009, Figure 2D). (Wu et al., 2017; Song et al., 2018; Yu, 2018; Luo, 2020). The certainty of the evidence for this outcome was assessed as low due to imprecision caused by a wide 95% CI and a small number of trials and patients, see Supplementary Table S1.

#### 3.4.2 Combined CHM-WM group *versus* WM alone group

Primary outcome

##### 3.4.2.1 Continuation of pregnancy after 28 gestational weeks

**FIGURE 3:**
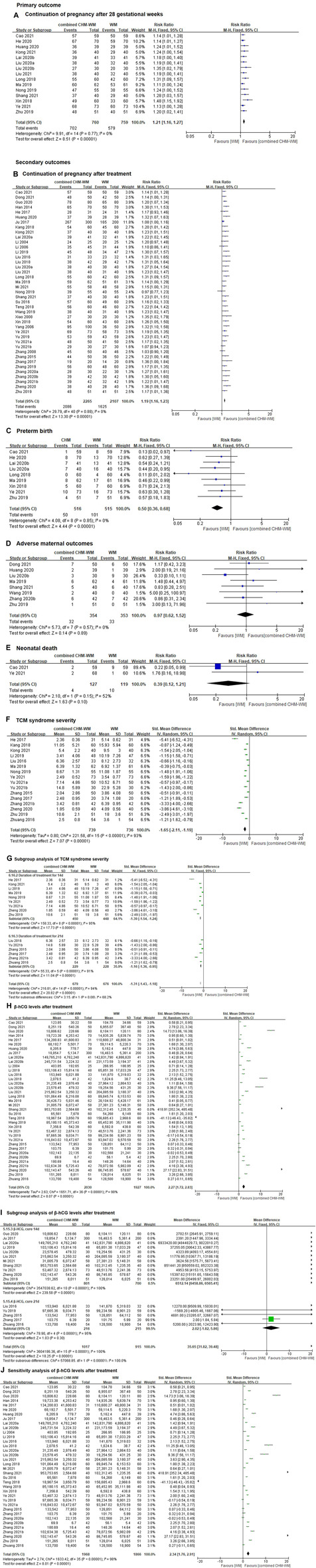
Forest plot of combined CHM-WM group versus WM alone group. **(A)** Continuation of pregnancy after 28 gestational weeks; **(B)** Continuation of pregnancy after treatment; Preterm birth; **(D)** Adverse maternal outcomes; Neonatal death; TCM syndrome severity; **(G)** Subgroup analysis of TCM syndrome severity; β-hCG levels after treatment; Subgroup analysis of β-hCG levels after treatment; Sensitivity analysis of β-hCG levels after treatment. **(C)** preterm birth; **(E)** neonatal death; **(F)** TCM syndrome severity; **(H)** β-hCG levels after treatment; **(I)** subgroup analysis of β-hCG levels after treatment; **(J)** sensitivity analysis of β-hCG levels after treatment.

In total, 15 clinical trials with 1,519 patients suggested that the incidence of continuation of pregnancy after 28 gestational weeks was significantly increased in combined CHM-WM group compared with WM alone group (RR 1.21, 95% CI 1.16 to 1.27, I^2^ = 0%, *p* < 0.00001, Figure 3A). [(Cao and Yan, 2021), (He, 2020), (Huang, 2020), (Kong et al., 2021), (Lai et al., 2020b), (Liu et al., 2020), (Liu, 2020), (Liu, 2021), (Long, 2018), (Ma, 2019), (Nong, 2019), (Shang et al., 2021), (Xin et al., 2018), (Ye and Tao, 2021), (Zhu and Wang, 2019)] The certainty of the evidence was assessed as moderate due to imprecision caused by small number of patients, see Supplementary Table S2.

Secondary outcomes

##### 3.4.2.2 Continuation of pregnancy after treatment

Forty-one clinical trials including 4,372 patients suggested that the incidence of continuation of pregnancy after treatment was significantly increased in combined CHM-WM compared with WM alone group (RR 1.19, 95% CI 1.16 to 1.23, I^2^ = 0%, *p* < 0.00001, Figure 3B). [(Cao and Yan, 2021), (Dong and Zhang, 2021), (Guo et al., 2020), (Han et al., 2014), (He, 2017), (Huang, 2020), (Ju, 2017), (Kang and Wang, 2018), (Kong et al., 2021), (Lai et al., 2020a), (Li et al., 2004), (Li et al., 2006), (Li, 2019), (Liu and Cui, 2016), (Liu, 2018), (Liu et al., 2020), (Liu, 2021), (Long, 2018), (Ma, 2019), (Mi et al., 2021), (Nong, 2019), (Shang et al., 2021), (Su, 2016), (Teng, 2018), (Wang and Niu, 2019), (Xiao, 2008), (Xin et al., 2018), (Yang, 2006), (Ye and Tao, 2021), (Yu et al., 2019), (Yu et al., 2021), (Yu and Jiang, 2021), (Xiao, 2008), (Zhang and Ding, 2015), (Zhang, 2017), (Zhang et al., 2019), (Zhang, 2020), (Zhang and Hou, 2020), (Zhang, 2021b), (Zheng et al., 2020), (Zhu and Wang, 2019)] The certainty of the evidence was assessed as moderate due to imprecision caused by small number of patients, see Supplementary Table S2.

##### 3.4.2.3 Preterm birth

Preterm birth rates were reported in nine trials including 1,031 patients, which showed the preterm birth rate was significantly reduced in combined CHM-WM group compared with WM alone group (RR 0.50, 95% CI 0.36 to 0.68, I^2^ = 0%, *p* < 0.00001, Figure 3C). [(Cao and Yan, 2021), (He, 2020), (Lai et al., 2020b), (Liu et al., 2020), (Long, 2018), (Ma, 2019), (Xin et al., 2018), (Ye and Tao, 2021), (Zhu and Wang, 2019)] The certainty of the evidence for this outcome was assessed as low due to imprecision caused by a wide 95% CI and a small number of trials and patients, see Supplementary Table S2.

##### 3.4.2.4 Adverse maternal outcomes

In total, eight trials including 707 patients reported no significant difference in reducing the incidence of adverse in combined CHM-WM group compared with WM alone group (RR 0.97, 95% CI 0.62 to 1.52, I^2^ = 0%; *p* = 0.89, Figure 3D). [(Dong and Zhang, 2021), (Huang, 2020), (Liu, 2020), (Ma, 2019), (Shang et al., 2021), (Wang and Niu, 2019), (Zhang and Hou, 2020), (Zhu and Wang, 2019)] The certainty of the evidence for this outcome was assessed as low due to imprecision caused by a wide 95% CI and a small number of trials and patients, see Supplementary Table S2. Detailed adverse outcomes of each study were shown in the summary of adverse events in the included studies, see Supplementary Table S3.

##### 3.4.2.5 Adverse neonatal outcomes

Only 1 trial reported neonatal malformation in combined CHM-WM group. (Ye and Tao, 2021).

##### 3.4.2.6 Neonatal death

Two trials including 246 patients reported neonatal death, of these, four neonatal death cases were reported in patients receiving combined CHM-WM and 10 neonatal death cases were reported in patients who received WM alone. No significant difference in reducing the incidence of neonatal death was found in combined CHM-WM group compared with WM alone group. (RR 0.39; 95% CI 0.12 to 1.21, I^2^ = 52%, *p* = 0.15, Figure 3E). (Cao and Yan, 2021; Ye and Tao, 2021) The certainty of the evidence for this outcome was assessed as low due to imprecision caused by a wide 95% CI and a small number of trials and patients, see Supplementary Table S2.

##### 3.4.2.7 TCM syndrome severity

Sixteen trials included 1,475 patients suggested that the TCM syndrome severity was significantly reduced in the combined CHM-WM group compared with WM alone group (SMD -1.65, 95% CI –2.11 to −1.19, I^2^ = 93%, *p* < 0.00001, Figure 3F). [(He, 2017), (Kang and Wang, 2018), (Kong et al., 2021), (Li, 2019), (Liu and Cui, 2016), (Ma, 2019), (Nong, 2019), (Ye and Tao, 2021), (Yu et al., 2021), (Yu and Jiang, 2021), (Zhang and Ding, 2015), (Zhang, 2017), (Zhang, 2021b), (Zheng et al., 2020), (Zhu and Wang, 2019), (Zhuang, 2016)] The reason for using continuous outcome was the same as we have discussed in the CHM alone *versus* WM alone section. The certainty of the evidence for this outcome was assessed as low due to imprecision caused by a wide 95% CI and a small number of trials and patients, see Supplementary Table S2.

Subgroup analysis

We decided to conduct a subgroup analysis to investigate the source of the heterogeneity in the TCM syndrome score. Although the duration of treatment showed a lower TCM syndrome score in combined CHM-WM group compared with WM alone group, the heterogeneity was still high (I^2^ = 94%, Figure 3G). Short-term treatment (one course only) and long-term treatment (more than one course) did not show any difference in reducing the TCM syndrome score (*p* = 0.08, Figure 3G). Subgroup analysis for other planned comparisons was not available.

##### 3.4.2.8 Improvement in β-hCG levels after treatment

Thirty-seven RCTs including 3,957 patients suggested that the level of β-hCG was significantly reduced in combined CHM-WM group compared with WM alone group after treatment (SMD 2.27, 95% CI 1.72 to 2.83, I^2^ = 98%, *p* < 0.00001, Figure 3H). [(Cao and Yan, 2021), (Dong and Zhang, 2021), (Guo et al., 2020), (Han et al., 2014), (He, 2017), (He, 2020), (Huang, 2020), (Ju, 2017), (Lai et al., 2020a), (Lai et al., 2020b), (Li et al., 2004), (Li, 2019), (Liu and Cui, 2016), (Liu, 2018), (Liu et al., 2020), (Liu, 2020), (Liu, 2021), (Long, 2018), (Ma, 2019), (Mi et al., 2021), (Shang et al., 2021), (Su, 2016), (Teng, 2018), (Wang and Niu, 2019), (Xin et al., 2018), (Ye and Tao, 2021), (Yu et al., 2019), (Yu et al., 2021), (Zhang and Ding, 2015), (Zhang, 2017), (Zhang, 2020), (Zhang and Hou, 2020), (Zhang, 2021a), (Zhang, 2021b), (Zheng et al., 2020), (Zhu and Wang, 2019), (Zhuang, 2016)] The reason for using continuous outcome was the same as we have discussed in the CHM alone *versus* WM alone section. The certainty of the evidence for this outcome was assessed as low due to imprecision caused by a wide 95% CI and a small number of trials and patients, see Supplementary Table S2.

Subgroup analysis

Duration of treatment showed a higher level of β-hCG in combined CHM-WM when compared to WM alone after treatment, but heterogeneity was still high (I^2^ = 100%, Figure 3I). Short-term treatment (one course only) had a higher level of β-hCG when compared to long-term treatment (more than one course) (*p* < 0.00001, Figure 3I). Subgroup analysis for other planned comparisons was not available.

Sensitivity analysis

Sensitivity analysis was carried out by removing one trial with a gestation week range from 7 to 28. However, the heterogeneity was still high (SMD 2.34, 95% CI 1.76 to 2.91, I^2^ = 98%, Figure 3J). (Ma, 2019).

### 3.5 GRADE certainty of the evidence

The certainty of the evidence for continuation of pregnancy after 28 gestational weeks and continuation of pregnancy after treatment were judged to be moderate. And the certainty of the evidence for adverse maternal outcomes, preterm birth, neonatal death, TCM syndrome severity and β-hCG levels were judged to be low. Downgrading of evidence was based on different serious limitations of the imprecision due to the wide 95% CI and a small number of trials and patients. Although high heterogeneity in TCM syndrome severity and β-hCG levels were reported in this meta-analysis, heterogeneity was inevitable. Because the TCM syndrome severity was recorded by TCM syndrome score, and the judgments were made by TCM practitioners. Besides, the level of β-hCG changed a lot during the first trimester of pregnancy and β-hCG itself varies greatly among different participants. As the trials have provided the reason for the inconsistency in TCM syndrome severity and β-hCG levels and it is not related to the nature of the results. Thus, we decided not to downgrade the certainty of inconsistency. See Supplementary Tables S1,S2 for the details of the GRADE assessment.

## 4 Discussion

### 4.1 Principal findings

CHM alone *versus* WM alone for threatened miscarriage

Eleven RCTs with 1,029 women were included in this comparison of this updated review. In the treatment of threatened miscarriage, CHM alone group showed significant benefits in improving continuation of pregnancy after 28 weeks of gestation, continuation of pregnancy after treatment, TCM syndrome severity and β-hCG levels compared with WM alone group. No trials in this comparison reported adverse maternal outcomes, adverse neonatal outcomes, preterm birth and neonatal death. Due to a small number of trials and lack of detailed information, it was impossible to carry out the planned subgroup analyses.

Combined CHM-WM *versus* WM alone for threatened miscarriage

Forty-six RCTs with 4,852 women were included in this comparison of this updated review. In the treatment of threatened miscarriage, combined CHM-WM group showed significant improvement in the incidence of continuation of pregnancy after 28 weeks of gestation, continuation of pregnancy after treatment, preterm birth, TCM syndrome severity and β-hCG levels compared with WM alone group. While no significant differences in reducing the incidence of adverse maternal outcomes and neonatal death were found in this comparison group. Only one trial reported adverse neonatal outcomes in combined CHM-WM group. As more RCTs were included in this comparison and showed combined CHM-WM was more effective, CHM therapy could be a potential treatment for threatened miscarriage.

Meta-analysis of β-hCG and TCM syndrome severity showed high heterogeneity, thus we carried out subgroup analysis and sensitivity analysis to identify the potential source of the heterogeneity. However, the heterogeneity did not decrease significantly. After analysis of the article, the reason for high heterogeneity might be: 1) The level of β-hCG varies greatly among different patients during the first trimester of pregnancy (gestational week <12 weeks). Studies published by other teams also showed high heterogeneity of β-hCG but without any further investigation for the cause of high heterogeneity; [(Li et al., 2021), (Salari et al., 2020)] 2) Different studies used different CHM prescription ingredients, doses and treatment duration. Therefore, high heterogeneity is inevitable among the included studies. The certainty of the evidence for the main outcomes was from moderate to low due to different serious limitations of the imprecision caused by the range of 95% CI and the number of trials and patients. As most of the included trials had different CHM formulations, dosage, and duration of therapy, making a uniform protocol for standard treatment may be difficult and it also violates the principle of TCM syndrome differentiation and treatment.

### 4.2 Comparison with existing literature

Compared with other published meta-analyses by different teams, the same conclusion (CHM alone or combined CHM-WM was more effective than WM alone for threatened miscarriage treatment) on CHM for threatened miscarriage was reported. [(Gao et al., 2016), (He et al., 2019)] However, they are all limited by poor methodologies, such as, only a small number of RCTs were included, and most of them were assessed at high risk of bias and published years ago. Besides, they mainly focus on the efficacy evaluation of CHM treatment and barely consider the safety assessment due to the limited data.

Comparisons between this updated review and our previously published review in 2012 are: 1) In our previous review, 44 RCTs were included for meta-analysis but only 11 trials reported the details of randomization methods, so the sample size and quality of the included studies are limited. Based on the limitation, in this updated review we only included studies with details of randomization methods, and in total 57 RCTs were included for meta-analysis, more rigorous methodology and qualified RCTs. Besides, all the included RCTs were reported at low risk of bias at intervention deviation, result selection and outcome measurement. Hence, the results of the updated review provided a higher quality of evidence and a credible conclusion to the clinical practice; 2) In our previous review, there was no laboratory data available for meta-analysis. In this updated review, we collected and compared the values of laboratory investigations, for example, improvement in β-hCG levels after treatment as a surrogate indicator of efficacy. In addition, we also included other outcome measures, including TCM syndrome severity as an important indicator to evaluate the efficacy of TCM based on Chinese medicine theory. Our updated review provided more comprehensive information and both laboratory data and TCM syndrome severity supported the efficacy of CHM in the treatment of threatened miscarriage; 3) In our previous review, no safety outcome was reported, thus we were unable to draw any conclusion about the safety of CHM for mothers and fetuses. In this updated review, we have included and compared the adverse outcomes, including adverse maternal outcomes and neonatal death. The results showed no significant difference between the combined CHM-WM group and WM alone group, suggesting that CHM is safe for pregnancy.

Therefore, based on the above advantages in this updated review, we have a larger sample size, more high-quality studies and stronger evidence to confirm both the efficacy and safety of CHM for threatened miscarriage to recommend CHM to be a potential alternative therapy for physicians and patients in the treatment of threatened miscarriage.

### 4.3 Strengths and limitations

The main strengths of our study included: 1) a rigorous methodology was conducted for performing the updated systematic review and meta-analysis; 2) the inclusion of a relatively large number of RCTs and most of which were recently published; 3) strict risk of bias assessment were conducted in all RCTs, which made the finding more persuasive and reliable; 4) A broad possible range of reported outcomes were included, which provide a comprehensive summary of relevant information to support the efficacy of CHM for threatened miscarriage; 5) Potential sources of heterogeneity were determined and discussed in details through several subgroup analyses and sensitivity analysis.

Several limitations need to be considered in the interpretation of our results. 1) High heterogeneity of TCM syndrome severity and β-hCG were reported, which have been discussed in the ‘GRADE certainty of the evidence’ section and ‘Principal findings’ section; 2) Chinese medicine practitioners would slightly modify the classical formula by adding or removing some CHM for better treatment effects according to the TCM syndrome differentiation and treatment principle. Therefore, outcome data were not reported according to the CHM formula, making relevant subgroup analysis impracticable. 3) Very few RCTs reported adverse events, due to the limited available data, planned sensitivity analysis could not be performed. Therefore, more high-quality RCTs are needed to provide sufficient information for the long-term study of CHM’s safety in treating threatened miscarriage.

## 5 Conclusion and implications

Worldwide, there is currently no effective treatment for threatened miscarriage. Our meta-analysis of 57 RCTs suggested that CHM could be a potential treatment for threatened miscarriage, given that it showed a significant immediate effect on continuation of pregnancy after treatment and improvement in a longer gestational stage after 28 weeks. Besides, no increased risk of adverse events was found in either the mother or the newborn in combined CHM-WM group compared with WM alone group, but this finding was partial and requires further evaluation. Furthermore, according to the reported studies and clinical evidence, it showed that CHM could be a potential intervention when given for the prevention of miscarriage [(Li et al., 2016), (Zhou et al., 2022), Therefore, our updated review recommended CHM therapy as an alternative for threatened miscarriage, as it can prevent miscarriage better, without increasing maternal and neonatal risk. However, long-term safety information is still lacking which requires further investigation. Therefore, larger sample size and RCTs with a low risk of bias are needed to facilitate the accumulation of high-certainty evidence and the translation of clinical practice.

## Data Availability

The original contributions presented in the study are included in the article/Supplementary Material, further inquiries can be directed to the corresponding authors.
