# Supplementary material for: Chinese herbal medicine for threatened miscarriage: An updated systematic review and meta-analysis
Source: Front Pharmacol. 2023 Feb 14;14:1083746. doi: 10.3389/fphar.2023.1083746 (PMC9971626; doi:10.3389/fphar.2023.1083746)
Supplement: Supplementary file 1 [file DataSheet1.ZIP › Appendix A. INPLASY-protocol.pdf]

INPLASY

International Platform of Registered Systematic Review and Meta-analysis Protocols

Copyright © 2020-2022 INPLASY® Published by INPLASY®

INPLASY20220107

Chinese herbal medicine for threatened miscarriage: a systematic review and meta-analysis

International Platform of Registered Systematic Review and Meta-analysis Protocols

INPLASY PROTOCOL

To cite: Xie et al. Chinese herbal medicine for threatened miscarriage: a systematic review and meta-analysis. Inplasy protocol 202260107. doi: 10.37766/inplasy2022.6.0107

Received: 28 June 2022

Published: 28 June 2022

Corresponding author:
Lu Li

luciali@zju.edu.cn

Author Affiliation:
Pharmaceutical Informatics Institute, College of Pharmaceutical Sciences, Zhejiang University, Hangzhou, 310058, China.

Support: QJD1602022; ZYYCXTD-D-202002.

Review Stage at time of this submission: Completed but not published.

Conflicts of interest: None declared.

Chinese herbal medicine for threatened miscarriage: a systematic review and meta-analysis

Xie, HL[1]; Zhang, AL[2]; Mou, X[3]; Wang, CC[4]; Fan, XH[5]; Li, L[6].

Review question / Objective: To review the therapeutic effects and safety of Chinese herbal medicine for the treatment of threatened miscarriage.

Condition being studied: Only Randomized controlled trials with explicit randomization method and compared CHM (alone or in combination with other pharmaceuticals) with placebo, no treatment (including bed rest), or other pharmaceuticals as treatments for threatened miscarriage will be included.

Information sources: 1. EMBASE (30 November 2021) 2. MEDLINE (30 November 2021) 3. PubMed (30 November 2021) 4. CENTRAL (30 November 2021) 5. China National Knowledge Infrastructure (CNKI) (30 November 2021) 6. WanFang Database (30 November 2021) 7. VIP database (30 November 2021).

INPLASY registration number: This protocol was registered with the International Platform of Registered Systematic Review and Meta-Analysis Protocols (INPLASY) on 28 June 2022 and was last updated on 28 June 2022 (registration number INPLASY202260107).

Xie et al. Inplasy protocol 202260107. doi:10.37766/inplasy2022.6.0107 Downloaded from https://inplasy.com/inplasy-2022-6-0107/

INTRODUCTION

Review question / Objective: To review the therapeutic effects and safety of Chinese herbal medicine for the treatment of threatened miscarriage.

Condition being studied: Only Randomized controlled trials with explicit randomization method and compared CHM (alone or in combination with other pharmaceuticals) with placebo, no treatment (including bed rest), or other pharmaceuticals as

treatments for threatened miscarriage will be included.

METHODS

Participant or population: Women with threatened miscarriage at or before 28 weeks' gestation, regardless of underlying causes were included.

Intervention: All types of CHM in either standard or combined regimens for the treatment of threatened miscarriage, regardless of the dose or duration of administration, were compared with placebo, no treatment or WM. We planned the following comparisons.· CHM versus placebo.· CHM versus no treatment (including bed rest).· CHM alone versus WM alone.· Combined CHM and WM (CHM-WM) versus WM alone.

Comparator: All types of CHM in either standard or combined regimens for the treatment of threatened miscarriage, regardless of the dose or duration of administration, were compared with placebo, no treatment or WM. We planned the following comparisons.· CHM versus placebo.· CHM versus no treatment (including bed rest).· CHM alone versus WM alone.· Combined CHM and WM (CHM-WM) versus WM alone.

Study designs to be included: Randomized controlled trials (RCTs) with low selection bias were included.

Eligibility criteria: Quasi-randomized, cluster-randomized trials, non-randomized and cross-over trials were excluded. There were no language restrictions among all the includedRCTs.

Information sources: 1. EMBASE (30 November 2021) 2. MEDLINE (30 November 2021) 3. PubMed (30 November 2021) 4. CENTRAL (30 November 2021) 5. China National Knowledge Infrastructure (CNKI) (30 November 2021) 6. WanFang Database (30 November 2021) 7. VIP database (30 November 2021).

Main outcome(s): Continuation of pregnancy after 28 weeks of gestation. Pregnancy after 28 weeks of gestation is generally considered viable, and miscarriage before 28 weeks is considered nonviable due to the extremely low birth weight and underdevelopment. In this review, only viable pregnancies and continuation of pregnancy after 28 weeks were considered as the primary outcome. The incidence of continuation of pregnancy after 28 weeks of gestation = (total cases-cases of miscarriage)/ total cases × 100%.

Quality assessment / Risk of bias analysis: Two review authors (Xie HL and Zhang AL) independently performed risk of bias using the criteria outlined in the Cochrane Handbook for Systematic Reviews of Interventions (Version 6.2) (Higgins 2021). It includes seven items as follows: sequence generation, blinding of participants, blinding of outcome assessors, allocation concealment, incomplete outcome data, selective outcome reporting, and other sources of bias. Each item was assessed as either low, unclear or high risk of bias.

Strategy of data synthesis: We used Review Manager software (RevMan 5.4.1, 2020) for statistical analysis. Risk ratio (RR) and 95% confidence interval were used to analyze the effect size of dichotomous data, and weight mean difference (WMD) was used to analyze the effect size of continuous data. Besides, we used the standard mean difference to eliminate inconsistencies in units of measurement and measurement variances. $\chi 2$ and I2 quantitative tests were used to test the heterogeneity among the studies. When $P < 0.10$, $I2 > 50\%$, it is suggested that there is heterogeneity between studies, and random-effect model was selected for meta-analysis, and when $P > 0.10$, $I2 < 50\%$, no obvious heterogeneity is suggested, and the fixed-effect model was selected for meta-analysis. We carried out subgroup analysis, sensitivity analysis or only descriptive analysis when heterogeneity was obvious.

Subgroup analysis: Significant heterogeneity was found in this review, however, no data were available to carry

Xie et al. Inplasy protocol 202260107. doi:10.37766/inplasy2022.6.0107 Downloaded from https://inplasy.com/inplasy-2022-6-0107/

out the following prespecified subgroup analyses. (1). Short-term treatment (one course only) versus long-term treatment (more than one course) (2). Early threatened miscarriage (gestational week ≤ 12) versus late threatened miscarriage (gestational week >12).

Sensitivity analysis: Sensitivity analyses were performed by excluding a study and analyzing the remaining data for each round to test the robustness of our results.

Language: There are no language limitations.

Country(ies) involved: China.

Keywords: Chinese herbal medicine; Western medicine, Threatened miscarriage; Randomized controlled trial; Systematic review; Meta-analysis; Efficacy; Safety.

Contributions of each author:

Author 1 - Hongliang Xie.
Email: xiehongliang2020@126.com
Author 2 - Aolin Zhang.
Email: 22119174@zju.edu.cn
Author 3 - Xuan Mou.
Email: 3170100133@zju.edu.cn
Author 4 - Chi Chiu Wang.
Email: ccwang@cuhk.edu.hk
Author 5 - Xiaohui Fan.
Email: fanxh@zju.edu.cn
Author 6 - Lu Li.
Email: luciali@zju.edu.cn

Xie et al. Inplasy protocol 202260107. doi:10.37766/inplasy2022.6.0107 Downloaded from https://inplasy.com/inplasy-2022-6-0107/

Keywords: Chinese herbal medicine; Western medicine,

Threatened miscarriage; Randomized controlled trial; Systematic review; Meta-analysis; Efficacy; Safety.

Download PDF - 202260107

Review Status: Completed but not published
