# Supplementary material for: Chinese herbal medicine for threatened miscarriage: An updated systematic review and meta-analysis
Source: Front Pharmacol. 2023 Feb 14;14:1083746. doi: 10.3389/fphar.2023.1083746 (PMC9971626; doi:10.3389/fphar.2023.1083746)
Supplement: Supplementary file 1 [file DataSheet1.ZIP › Appendix C. Search strategy-.docx]

**Appendix C.**

Search

*China National Knowledge Infrastructure (CNKI)*

1. SU % ' Chinese medicine ' OR SU % ' Traditional Chinese medicine ' OR SU % ' ethnic

medicine ')

2. SU % 'threatened abortion ' OR SU % ' threatened miscarriage '

3.1 AND 2

4. TKA % ' randomized controlled trials ' OR TKA % ' clinical trials '

5. 3 AND 4

6. publication time between (2012-02-01,2022-06-30)

7. 5 AND 6

*WanFang Database*

1. Chinese medicine OR (traditional Chinese medicine) OR (Chinese herbal medicine)

2. application OR (clinical use) OR therapy

3. 1 AND 2

4. miscarriage OR abortion

5. (threatened abortion) OR (threatened miscarriage)

6. 4 AND 5

7. 3 AND 6

8. ("2012/02/01"[Date - Publication]: "2022/06/30"[Date - Publication])

9. 7 AND 8

*VIP*

1. M = Chinese herbal medicine OR Chinese medicine OR Traditional Chinese medicine OR ethnic medicine

2. M = threatened abortion OR threatened miscarriage

3. 1 AND 2

4. R = effectiveness OR safety OR efficacy OR treatment OR clinical trial OR =

randomized controlled trial

5. 3 AND 4

6. ("2012/02/01"[Date - Publication]: "2022/06/30"[Date - Publication])

7. 5 AND 6

*PubMed*

1. "Medicine, Chinese Traditional"[Mesh]

2. (((("Traditional Chinese Medicine") [All Fields] OR ("Traditional Medicine, Chinese") [All Fields]) OR ("Chinese Traditional Medicine") [All Fields]) OR ("Chinese Medicine, Traditional") [All Fields]) OR ("Drugs, Chinese Herbal") [All Fields]

3. 1 AND 2

4. "Abortion, Threatened"[MeSH Terms]

5. (((("Threatened Miscarriages") [All Fields] OR (Threatened Abortions) [All Fields]) OR (Threatened Miscarriage) [All Fields]) OR (Miscarriage, Threatened) [All Fields]) OR (Threatened Abortion) [All Fields]

6. 4 AND 5

7. 3 AND 6

8. ("2012/02/01"[Date - Publication] : "2022/06/30"[Date - Publication])

9. 7 AND 8

*Cochrane Pregnancy and Childbirth’s Trials Register （CPC search* *is maintained by their Information Specialist）*

1. monthly searches of the Cochrane Central Register of Controlled Trials (CENTRAL)

which includes centralized searches of ClinicalTrials.gov and the WHO ICTRP

2. weekly searches of MEDLINE (Ovid)

3. weekly searches of Embase (Ovid)

4. monthly searches of CINAHL (EBSCO)

5. hand searches of 30 journals and the proceedings of major conferences

6. weekly current awareness alerts for a further 44 journals plus monthly BioMed Central email alerts.

*Central*

1. (chinese medicine):ti,ab,kw OR (traditional chinese medicine):ti,ab,kw OR (chinese

herbal medicine):ti,ab,kw

2. (threatened miscarriage):ti,ab,kw OR (threatened abortions):ti,ab,kw

3. Publication Year from 20120201 to 20220630

*Embase*

1. ('chinese medicine':ab,ti OR 'traditional chinese medicine':ab,ti OR 'chinese herbal

medicine':ab,ti)

2. ('threatened miscarriage':ab,ti OR 'threatened abortions':ab,ti)

3. 1 AND 2

4. [2012-2022]/py

5. 3 AND 4

*MEDLINE*

1. SU chinese medicine OR SU chinese herbal medicine OR SU traditional chinese

medicine

2. SU threatened miscarriage OR SU threatened abortions

3. [20120201-20220630]
