# Supplementary material for: Chinese herbal medicine for threatened miscarriage: An updated systematic review and meta-analysis
Source: Front Pharmacol. 2023 Feb 14;14:1083746. doi: 10.3389/fphar.2023.1083746 (PMC9971626; doi:10.3389/fphar.2023.1083746)
Supplement: Supplementary file 1 [file DataSheet1.ZIP › Appendix D. The botanical drugs of Chinese formulae in included RCTs-12.4.docx]

| **Combined CHM-WM versus WM alone.** | | | | |
| --- | --- | --- | --- | --- |
| **No.** | **Study ID** | **Prescription/**  **(Chinese formulae)** | **Raw material of botanical drugs** | **Preparation or details** |
| 1 | Kun Cao, 2021 | Jiawei Shoutai pill | *Cuscuta chinensis* Lam. [Convolvulaceae; Cuscutae semen], 20 g;  *Astragalus* *membranaceus* (Fisch.) Bge.var.*mongholicu*s (Bge.) Hsiao [Fabaceae; Astragali radix], 20 g;  *Dipsacus asper* Wall. ex Henry [Dipsacaceae; Dipsaci radix], 20g;  *Pseudostellaria* *heterophylla* (Miq.) Pax ex Pax et Hoffm. [Caryophyllaceae; Pseudostellariae radix], 15 g;  *Amomum* *villosum* Lour. [Zingiberaceae; Amomi fructus], 10 g;  *Astragalus* *membranaceus* (Fisch.) Bge.var.*mongholicus* (Bge.) Hsiao [Fabaceae; Astragali radix], 10 g;  *Taxillus* *chinensis* (DC.) Danser [Loranthaceae; Taxilli herba], 10 g;  *Equus* *asinus* L. [Equidae; Asini corii colla], 10 g;  *Atractylodes* *macrocephala* Koidz. [Asteraceae; Atractylodis macrocephalae rhizoma], 10 g;  *Glycyrrhiza* *uralensis* Fisch. [Fabaceae; Glycyrrhizae radix et rhizoma], 5 g. | Administration: p.o.  Dose: Water extraction  Dosing: Bid.  Duration: 20 days  The detailed extraction procedure was not reported in the manuscript. |
| 2 | Rong Dong 2021 | Zishen Yutai pill | *Cuscuta* *chinensis* Lam. [Convolvulaceae; Cuscutae semen];  *Amomum* *villosum* Lour. [Zingiberaceae; Amomi fructus];  *Rehmannia* *glutinosa* Libosch. [Scrophulariaceae; Rehmanniae radix praeparata];  *Panax* *ginseng* C.A.Mey. [Araliaceae; Ginseng radix et rhizoma];  *Taxillus* *chinensis* (DC.) Danser [Loranthaceae; Taxilli herba];  *Equus* *asinus* L. [Equidae; Asini corii colla];  *Polygonum* *multiflorum* Thunb. [Polygonaceae; Polygoni multiflori radix];  *Artemisia* *argyi* Levl.et Vant. [Asteraceae; Artemisiae argyi folium];  *Morinda* *officinalis* How [Rubiaceae; Morindae officinalis radix];  *Atractylodes* *macrocephala* Koidz. [Asteraceae; Atractylodis macrocephalae rhizoma];  *Codonopsis* *pilosula* (Franch.) Nannf. [Campanulaceae; Codonopsis radix]; | Administration: p.o.  Dose: 5 g of pills  Dosing: Tid.  Duration: 14 days  The detailed extraction procedure was not reported in the manuscript. |

|  |  |  | *Cervus* *elaphus* Linnaeus [Cervidae; Cervi cornu degelatinatum];  *Lycium* *barbarum* L. [Solanaceae; Lycii fructus];  *Dipsacus* *asper* Wall. ex Henry [Dipsacaceae; Dipsaci radix];  *Eucommia* *ulmoides* Oliv. [Eucommiaceae; Eucommiae cortex].  The amount of each drug in a polyherbal preparation is unavailable in the original text. |  |
| --- | --- | --- | --- | --- |
| 3 | Yan Guo, 2020 | Xionggui Jiaoai decoction | *Equus* *asinus* L. [Equidae; Asini corii colla], 18 g;  *Angelica* *sinensis* (Oliv.) Diels [Apiaceae; Angelicae sinensis radix], 18 g;  *Ligusticum* *chuanxiong* Hort. [Apiaceae; Chuanxiong rhizoma], 10 g;  *Glycyrrhiza* *uralensis* Fisch. [Fabaceae; Glycyrrhizae radix et rhizoma], 5 g;  *Artemisia* *argyi* Levl.et Vant. [Asteraceae; Artemisiae argyi folium], 30 g;  *Paeonia* *lactiflora* Pall. [Ranunculaceae Juss.; Paeoniae radix alba], 30 g;  *Rehmannia* *glutinosa* Libosch. [Scrophulariaceae; Rehmanniae radix], 20 g. | Administration: p.o.  Dose: 400 mL of water extraction  Dosing: Q.d.  Duration: 14 days  The detailed extraction procedure was not reported in the manuscript. |
| 4 | Chunyan Han, 2014 | Gushen Antai pill | *Polygonum* *multiflorum* Thunb. [Polygonaceae; Polygoni multiflori radix];  *Rehmannia* *glutinosa* Libosch. [Scrophulariaceae; Rehmanniae radix praeparata];  *Cistanche* *deserticola* Ma [Orobanchaceae; Cistanches herba];  *Dipsacus* *asper* Wall. ex Henry [Dipsacaceae; Dipsaci radix];  *Taxillus* *chinensis* (DC.) Danser [Loranthaceae; Taxilli herba];  *Uncaria* *rhynchophylla* (Miq.) Miq. ex Havil. [Rubiaceae; Uncariae ramulus cum uncis];  *Cuscuta* *chinensis* Lam. [Convolvulaceae; Cuscutae semen];  *Atractylodes* *macrocephala* Koidz. [Asteraceae; Atractylodis macrocephalae rhizoma];  *Scutellaria* *baicalensis* Georgi [Lamiaceae; Scutellariae radix];  *Paeonia* *lactiflora* Pall. [Ranunculaceae Juss.; Paeoniae radix alba].  The amount of each drug in a polyherbal preparation is unavailable in the original text. | Administration: p.o.  Dose: 1 bag of pills  Dosing: Tid.  Duration: 14 days  The detailed extraction procedure was not reported in the manuscript. |
| 5 | Lijie He, 2017 | Shoutai Yigong powder | *Agrimonia* *pilosa* Ledeb. [Rosaceae; Agrimoniae herba], 30 g;  *Adenophora* *tetraphylla* (Thunb.) Fisch. [Campanulaceae; Adenophorae radix], 30 g;  *Cuscuta* *chinensis* Lam. [Convolvulaceae; Cuscutae semen], 20 g;  *Dipsacus* *asper* Wall. ex Henry [Dipsacaceae; Dipsaci radix], 20 g;  *SepielLa* *maindroni* *de* Rochebrune [Sepiidae; Sepiae endoconcha], 20 g;  *Taxillus* *chinensis* (DC.) Danser [Loranthaceae; Taxilli herba], 15g;  *Poria* *cocos* (Schw.) Wolf [Polyporaceae; Poria], 10 g;  *Citrus* *reticulata* Blanco [Rutaceae; Citri reticulatae pericarpium], 10 g;  *Atractylodes* *macrocephala* Koidz. [Asteraceae; Atractylodis macrocephalae rhizoma], 10 g;  *Rubia* *cordifolia* L. [Rubiaceae; Rubiae radix et rhizoma], 10 g;  *Glycyrrhiza* *uralensis* Fisch. [Fabaceae; Glycyrrhizae radix et rhizoma], 6 g. | Administration: p.o.  Dose: Water extraction  Dosing: Bid.  Duration: 10 days  The detailed extraction procedure was not reported in the manuscript. |
| 6 | Pei He, 2020 | Guben Antai decoction | *Cuscuta* *chinensis* Lam. [Convolvulaceae; Cuscutae semen], 15 g;  *Codonopsis* *pilosula* (Franch.) Nannf. [Campanulaceae; Codonopsis radix], 15 g;  *Equus* *asinus* L. [Equidae; Asini corii colla], 10 g;  *Taxillus* *chinensis* (DC.) Danser [Loranthaceae; Taxilli herba], 10 g;  *Dipsacus* *asper* Wall. ex Henry [Dipsacaceae; Dipsaci radix], 10 g;  *Atractylodes* *macrocephala* Koidz. [Asteraceae; Atractylodis macrocephalae rhizoma], 10 g;  *Scutellaria* *baicalensis* Georgi [Lamiaceae; Scutellariae radix], 10 g;  *Paeonia* *lactiflora* Pall. [Ranunculaceae Juss.; Paeoniae radix alba], 10 g;  *Artemisia* *argyi* Levl.et Vant. [Asteraceae; Artemisiae argyi folium], 6 g;  *Amomum* *villosum* Lour. [Zingiberaceae; Amomi fructus], 3 g. | Administration: p.o.  Dose: 200 mL of water extraction  Dosing: Bid.  Duration: 14 days  The detailed extraction procedure was not reported in the manuscript. |
| 7 | Ling Huang, 2020 | Gushen Antai pill | *Polygonum* *multiflorum* Thunb. [Polygonaceae; Polygoni multiflori radix];  *Rehmannia* *glutinosa* Libosch. [Scrophulariaceae; Rehmanniae radix];  *Cistanche* *deserticola* Ma [Orobanchaceae; Cistanches herba];  *Dipsacus* *asper* Wall. ex Henry [Dipsacaceae; Dipsaci radix];  *Taxillus* *chinensis* (DC.) Danser [Loranthaceae; Taxilli herba];  *Uncaria* *rhynchophylla* (Miq.) Miq. ex Havil. [Rubiaceae; Uncariae ramulus cum uncis];  *Cuscuta* *chinensis* Lam. [Convolvulaceae; Cuscutae semen];  *Atractylodes* *macrocephala* Koidz. [Asteraceae; Atractylodis macrocephalae rhizoma];  *Scutellaria* *baicalensis* Georgi [Lamiaceae; Scutellariae radix];  *Paeonia* *lactiflora* Pall. [Ranunculaceae Juss.; Paeoniae radix alba].  The amount of each drug in a polyherbal preparation is unavailable in the original text. | Gushen Antai Pill (SFDA approval No.: Z20030144)  Beijing Bran Pharmaceutical Co.  Administration: p.o.  Dose: 6 g of pills  Dosing: Tid.  Duration: 14 days  The detailed extraction procedure was not reported in the manuscript. |
| 8 | Jie Ju 2017 | Gushen Antai pill | *Polygonum* *multiflorum* Thunb. [Polygonaceae; Polygoni multiflori radix];  *Rehmannia* *glutinosa* Libosch. [Scrophulariaceae; Rehmanniae radix];  *Cistanche* *deserticola* Ma [Orobanchaceae; Cistanches herba];  *Dipsacus* *asper* Wall. ex Henry [Dipsacaceae; Dipsaci radix];  *Taxillus* *chinensis* (DC.) Danser [Loranthaceae; Taxilli herba];  *Uncaria* *rhynchophylla* (Miq.) Miq. ex Havil. [Rubiaceae; Uncariae ramulus cum uncis];  *Cuscuta* *chinensis* Lam. [Convolvulaceae; Cuscutae semen];  *Atractylodes* *macrocephala* Koidz. [Asteraceae; Atractylodis macrocephalae rhizoma];  *Scutellaria* *baicalensis* Georgi [Lamiaceae; Scutellariae radix];  *Paeonia* *lactiflora* Pall. [Ranunculaceae Juss.; Paeoniae radix alba].  The amount of each drug in a polyherbal preparation is unavailable in the original text. | Administration: p.o.  Dose: 1 bag of pills  Dosing: Tid.  Duration: 14 days  The detailed extraction procedure was not reported in the manuscript. |
| 9 | Yangfang, Kang, 2021 | Yangxue Antai decoction | *Scutellaria* *baicalensis* Georgi [Lamiaceae; Scutellariae radix], 15 g;  *Codonopsis* *pilosula* (Franch.) Nannf. [Campanulaceae; Codonopsis radix], 15 g;  *Atractylodes* *macrocephala* Koidz. [Asteraceae; Atractylodis macrocephalae rhizoma], 15 g;  *Paeonia* *lactiflora* Pall. [Ranunculaceae Juss.; Paeoniae radix alba], 15 g;  *Angelica* *sinensis* (Oliv.) Diels [Apiaceae; Angelicae sinensis radix], 10 g;  *Rehmannia* *glutinosa* Libosch. [Scrophulariaceae; Rehmanniae radix], 10 g;  *Rehmannia* *glutinosa* Libosch. [Scrophulariaceae; Rehmanniae radix praeparata], 10 g;  *Equus* *asinus* L. [Equidae; Asini corii colla], 15 g;  *Taxillus* *chinensis* (DC.) Danser [Loranthaceae; Taxilli herba], 15 g;  *Dipsacus* *asper* Wall. ex Henry [Dipsacaceae; Dipsaci radix], 15 g;  *Astragalus* *membranaceus* (Fisch.) Bge.var.*mongholicus* (Bge.) Hsiao [Fabaceae; Astragali radix], 6 g;  *Amomum* *villosum* Lour. [Zingiberaceae; Amomi fructus], 10 g;  *Eucommia* *ulmoides* Oliv. [Eucommiaceae; Eucommiae cortex], 10 g;  *Glycyrrhiza* *uralensis* Fisch. [Fabaceae; Glycyrrhizae radix et rhizoma], 6 g. | Administration: p.o.  Dose: Water extraction  Dosing: Bid.  Duration: 14 days  The detailed extraction procedure was not reported in the manuscript. |
| 10 | Dejia Kong, 2021 | Bushen Huoxue recipe | *Angelica* *sinensis* (Oliv.) Diels [Apiaceae; Angelicae sinensis radix], 9 g;  *Salvia* *miltiorrhiza* Bge. [Lamiaceae; Salviae miltiorrhizae radix et rhizoma], 10 g;  *Panax* *notoginseng* (Burk.) F. H. Chen [Araliaceae; Notoginseng radix et rhizoma], 3 g;  *Boehmeria* *nivea* (L.) Gaudich. [Urticaceae; Radix boehmeriae], 20 g;  *Taxillus* *chinensis* (DC.) Danser [Loranthaceae; Taxilli herba], 12 g;  *Dipsacus* *asper* Wall. ex Henry [Dipsacaceae; Dipsaci radix], 12 g;  *Cuscuta* *chinensis* Lam. [Convolvulaceae; Cuscutae semen], 20 g;  *Astragalus* *membranaceus* (Fisch.) Bge.var.*mongholicus* (Bge.) Hsiao [Fabaceae; Astragali radix], 12 g;  *Scutellaria* *baicalensis* Georgi [Lamiaceae; Scutellariae radix], 9 g;  *Equus* *asinus* L. [Equidae; Asini corii colla], 9 g;  *Glycyrrhiza* *uralensis* Fisch. [Fabaceae; Glycyrrhizae radix et rhizoma], 5 g. | Administration: p.o.  Dose: Water extraction  Dosing: Bid.  Duration: 14 days  The detailed extraction procedure was not reported in the manuscript. |
| 11 | Juan Lai, 2020 | Chinese medicine formula | *Pseudostellaria* *heterophylla* (Miq.) Pax ex Pax et Hoffm. [Caryophyllaceae; Pseudostellariae radix], 10 g;  *Eucommia* *ulmoides* Oliv. [Eucommiaceae; Eucommiae cortex], 10 g;  *Dipsacus* *asper* Wall. ex Henry [Dipsacaceae; Dipsaci radix], 10 g;  *Paeonia* *lactiflora* Pall. [Ranunculaceae Juss.; Paeoniae radix alba], 10 g;  *Cuscuta* *chinensis* Lam. [Convolvulaceae; Cuscutae semen], 10 g;  *Dioscorea* *opposita* Thunb. [Dioscoreaceae; Dioscoreae rhizoma], 15 g;  *Rehmannia* *glutinosa* Libosch. [Scrophulariaceae; Rehmanniae radix praeparata], 10 g;  *Atractylodes* *macrocephala* Koidz. [Asteraceae; Atractylodis macrocephalae rhizoma], 10 g;  *Boehmeria* *nivea* (L.) Gaudich. [Urticaceae; Radix boehmeriae], 15 g;  *Codonopsis* *pilosula* (Franch.) Nannf. [Campanulaceae; Codonopsis radix]. 10 g;  *Astragalus* *membranaceus* (Fisch.) Bge.var.*mongholicus* (Bge.) Hsiao [Fabaceae; Astragali radix], 10 g;  *Agrimonia* *pilosa* Ledeb. [Rosaceae; Agrimoniae herba], 15 g;  *Glycyrrhiza* *uralensis* Fisch. [Fabaceae; Glycyrrhizae radix et rhizoma], 2 g. | Administration: p.o.  Dose: Water extraction  Dosing: Bid.  Duration: 14 days  The detailed extraction procedure was not reported in the manuscript. |
| 12 | Li Lai, 2020 | Shoutai pill | *Cuscuta* *chinensis* Lam. [Convolvulaceae; Cuscutae semen], 20 g;  *Taxillus* *chinensis* (DC.) Danser [Loranthaceae; Taxilli herba], 10 g;  *Dipsacus* *asper* Wall. ex Henry [Dipsacaceae; Dipsaci radix], 15 g;  *Equus* *asinus* L. [Equidae; Asini corii colla], 10 g;  *Pseudostellaria* *heterophylla* (Miq.) Pax ex Pax et Hoffm. [Caryophyllaceae; Pseudostellariae radix], 20 g;  *Astragalus* *membranaceus* (Fisch.) Bge.var.*mongholicus* (Bge.) Hsiao [Fabaceae; Astragali radix], 20 g;  *Atractylodes* *macrocephala* Koidz. [Asteraceae; Atractylodis macrocephalae rhizoma], 10 g;  *Scutellaria* *baicalensis* Georgi [Lamiaceae; Scutellariae radix], 10 g;  *Ligustrum* *lucidum* Ait. [Oleaceae; Ligustri lucidi fructus], 15 g;  *Eclipta* *prostrata* L. [Asteraceae; Ecliptae herba], 15 g;  *Glycyrrhiza* *uralensis* Fisch. [Fabaceae; Glycyrrhizae radix et rhizoma]. 5 g. | Administration: p.o.  Dose: 300 mL of water extraction  Dosing: Bid.  Duration: 20 days  The detailed extraction procedure was not reported in the manuscript. |
| 13 | Weili Li 2004 | Bushen Antai decoction | *Cuscuta chinensis* Lam. [Convolvulaceae; Cuscutae semen], 10 g;  *Eucommia ulmoides* Oliv. [Eucommiaceae; Eucommiae cortex], 10 g;  *Taxillus chinensis* (DC.) Danser [Loranthaceae; Taxilli herba], 10 g;  *Dipsacus asper* Wall. ex Henry [Dipsacaceae; Dipsaci radix], 10 g;  *Pseudostellaria heterophylla* (Miq.) Pax ex Pax et Hoffm. [Caryophyllaceae; Pseudostellariae radix], 10 g;  *Astragalus membranaceus* (Fisch.) Bge.var.*mongholicus* (Bge.) Hsiao [Fabaceae; Astragali radix], 10 g;  *Atractylodes macrocephala* Koidz. [Asteraceae; Atractylodis macrocephalae rhizoma], 10 g;  *Scutellaria baicalensis* Georgi [Lamiaceae; Scutellariae radix], 10 g;  *Paeonia lactiflora* Pall. [Ranunculaceae Juss.; Paeoniae radix alba], 10 g;  *Rehmannia glutinosa* Libosch. [Scrophulariaceae; Rehmanniae radix praeparata], 10 g;  *Boehmeria nivea* (L.) Gaudich. [Urticaceae; Radix boehmeriae], 10 g. | Administration: p.o.  Dose: Water extraction  Dosing: Bid.  Duration: 10 days  The detailed extraction procedure was not reported in the manuscript. |
| 14 | Lin Li 2006 | Bushen Gutai decoction | *Cuscuta chinensis* Lam. [Convolvulaceae; Cuscutae semen], 30 g;  Lycium barbarum L. [Solanaceae; Lycii fructus], 30 g;  *Taxillus chinensis* (DC.) Danser [Loranthaceae; Taxilli herba], 20 g;  *Dipsacus asper* Wall. ex Henry [Dipsacaceae; Dipsaci radix], 20 g;  *Codonopsis pilosula* (Franch.) Nannf. [Campanulaceae; Codonopsis radix], 30 g;  *Dioscorea opposita* Thunb. [Dioscoreaceae; Dioscoreae rhizoma], 15 g;  *Eucommia ulmoides* Oliv. [Eucommiaceae; Eucommiae cortex], 20 g;  *Paeonia lactiflora* Pall. [Ranunculaceae Juss.; Paeoniae radix alba], 20 g;  *Glycyrrhiza uralensis* Fisch. [Fabaceae; Glycyrrhizae radix et rhizoma], 5 g. | Administration: p.o.  Dose: 400 mL of water extraction  Dosing: Bid.  Duration: 10 days  The detailed extraction procedure was not reported in the manuscript. |
| 15 | Hui Li, 2019 | Chushi Antai recipe | *Dipsacus* *asper* Wall. ex Henry [Dipsacaceae; Dipsaci radix], 15 g;  *Eucommia* *ulmoides* Oliv. [Eucommiaceae; Eucommiae cortex], 15 g;  *Cuscuta* *chinensis* Lam. [Convolvulaceae; Cuscutae semen], 15 g;  *Pseudostellaria* *heterophylla* (Miq.) Pax ex Pax et Hoffm. [Caryophyllaceae; Pseudostellariae radix], 15 g;  *Atractylodes* *macrocephala* Koidz. [Asteraceae; Atractylodis macrocephalae rhizoma], 15 g;  *Scutellaria* *baicalensis* Georgi [Lamiaceae; Scutellariae radix], 10 g;  *Eclipta* *prostrata* L. [Asteraceae; Ecliptae herba], 10 g;  *Equus* *asinus* L. [Equidae; Asini corii colla], 10 g;  *Perilla* *frutescens* (L.) Britton [Lamiaceae; Perillae caulis], 10 g;  *Amomum* *villosum* Lour. [Zingiberaceae; Amomi fructus], 10 g;  *Glycyrrhiza* *uralensis* Fisch. [Fabaceae; Glycyrrhizae radix et rhizoma], 10 g. | Administration: p.o.  Dose: 200 mL of water extraction  Dosing: Bid.  Duration: 14 days  The detailed extraction procedure was not reported in the manuscript. |
| 16 | Xiaofeng Liu, 2016 | Bushen Jianpi recipe combined with acupoint sticking therapy | Bushen Jianpi recipe:  *Codonopsis* *pilosula* (Franch.) Nannf. [Campanulaceae; Codonopsis radix], 18 g;  *Astragalus* *membranaceus* (Fisch.) Bge.var.*mongholicus* (Bge.) Hsiao [Fabaceae; Astragali radix], 18 g;  *Taxillus* *chinensis* (DC.) Danser [Loranthaceae; Taxilli herba], 15 g;  *Cuscuta* *chinensis* Lam. [Convolvulaceae; Cuscutae semen], 15 g;  *Eucommia* *ulmoides* Oliv. [Eucommiaceae; Eucommiae cortex], 15 g;  *Dipsacus* *asper* Wall. ex Henry [Dipsacaceae; Dipsaci radix], 15 g;  *Paeonia* *lactiflora* Pall. [Ranunculaceae Juss.; Paeoniae radix alba], 15 g;  *Atractylodes* *macrocephala* Koidz. [Asteraceae; Atractylodis macrocephalae rhizoma], 12 g;  *Scutellaria* *baicalensis* Georgi [Lamiaceae; Scutellariae radix], 12 g;  *Cyperus* *rotundus* L. [Cyperaceae; Cyperi rhizome], 12 g;  *Glycyrrhiza* *uralensis* Fisch. [Fabaceae; Glycyrrhizae radix et rhizoma], 6 g.  Acupoint sticking therapy:  *Cuscuta* *chinensis* Lam. [Convolvulaceae; Cuscutae semen];  *Taxillus* *chinensis* (DC.) Danser [Loranthaceae; Taxilli herba];  *Dipsacus* *asper* Wall. ex Henry [Dipsacaceae; Dipsaci radix].  The amount of each drug in a polyherbal preparation is unavailable in original text. | Bushen Jianpi recipe:  Administration: p.o.  Dose: Water extraction  Dosing: Bid.  Duration: 21 days  The detailed extraction procedure was not reported in the manuscript.  Acupoint sticking therapy:  Administration: p.o.  Dose: 1 patch  Dosing: Q.d.  Duration: 4h |
| 17 | Fenghua Liu, 2018 | Bushen Yiqi Zhitong Antai recipe | *Salvia* *miltiorrhiza* Bge. [Lamiaceae; Salviae miltiorrhizae radix et rhizoma], 12 g;  *Psoralea* *corylifolia* L. [Fabaceae; Psoraleae fructus], 12 g;  *Atractylodes* *macrocephala* Koidz. [Asteraceae; Atractylodis macrocephalae rhizoma], 9 g;  *Glycyrrhiza* *uralensis* Fisch. [Fabaceae; Glycyrrhizae radix et rhizoma], 9 g;  *Ligustrum* *lucidum* Ait. [Oleaceae; Ligustri lucidi fructus], 9 g;  *Paeonia* *lactiflora* Pall. [Ranunculaceae Juss.; Paeoniae radix alba], 20 g;  *Taxillus* *chinensis* (DC.) Danser [Loranthaceae; Taxilli herba], 20 g;  *Astragalus* *membranaceus* (Fisch.) Bge.var.*mongholicus* (Bge.) Hsiao [Fabaceae; Astragali radix], 20 g;  *Cucurbita* *moschata* (Duchesne ex Lam.) Duchesne ex Poir. [Cucurbitaceae; Pedicellus cucurbitae], 20 g;  *Cuscuta* *chinensis* Lam. [Convolvulaceae; Cuscutae semen], 30 g;  *Eclipta* *prostrata* L. [Asteraceae; Ecliptae herba], 15 g. | Administration: p.o.  Dose: Water extraction  Dosing: Bid.  Duration: 14 days  The detailed extraction procedure was not reported in the manuscript. |
| 18 | Fengping Liu, 2020 | Guben Antai decoction | *Codonopsis* *pilosula* (Franch.) Nannf. [Campanulaceae; Codonopsis radix], 15 g;  *Equus* *asinus* L. [Equidae; Asini corii colla], 10 g;  *Atractylodes* *macrocephala* Koidz. [Asteraceae; Atractylodis macrocephalae rhizoma], 10 g;  *Scutellaria* *baicalensis* Georgi [Lamiaceae; Scutellariae radix], 10 g;  *Taxillus* *chinensis* (DC.) Danser [Loranthaceae; Taxilli herba], 10 g;  *Cuscuta* *chinensis* Lam. [Convolvulaceae; Cuscutae semen], 15 g;  *Dipsacus* *asper* Wall. ex Henry [Dipsacaceae; Dipsaci radix], 10 g;  *Artemisia* *argyi* Levl.et Vant. [Asteraceae; Artemisiae argyi folium], 6 g;  *Amomum* *villosum* Lour. [Zingiberaceae; Amomi fructus], 6 g. | Administration: p.o.  Dose: Water extraction  Dosing: Bid.  Duration: 14 days  The detailed extraction procedure was not reported in the manuscript. |
| 19 | Hongxia Liu, 2020 | Antai pill | *Cuscuta* *chinensis* Lam. [Convolvulaceae; Cuscutae semen], 18 g;  *Astragalus* *membranaceus* (Fisch.) Bge.var.*mongholicus* (Bge.) Hsiao [Fabaceae; Astragali radix], 15 g;  *Dioscorea* *opposita* Thunb. [Dioscoreaceae; Dioscoreae rhizoma], 15 g;  *Taxillus* *chinensis* (DC.) Danser [Loranthaceae; Taxilli herba], 15 g;  *Atractylodes* *macrocephala* Koidz. [Asteraceae; Atractylodis macrocephalae rhizoma], 15 g;  *Dipsacus* *asper* Wall. ex Henry [Dipsacaceae; Dipsaci radix], 12 g;  *Codonopsis* *pilosula* (Franch.) Nannf. [Campanulaceae; Codonopsis radix], 12 g;  *Equus* *asinus* L. [Equidae; Asini corii colla], 10 g;  *Paeonia* *lactiflora* Pall. [Ranunculaceae Juss.; Paeoniae radix alba], 10 g. | Antai pill (SFDA approval No.: Z20920030180)  Administration: p.o.  Dose: 6 g of pills  Dosing: Bid.  Duration: 14 days  The detailed extraction procedure was not reported in the manuscript. |
| 20 | Chundi Liu, 2021 | Zishen Yangtai recipe | *Pseudostellaria* *heterophylla* (Miq.) Pax ex Pax et Hoffm. [Caryophyllaceae; Pseudostellariae radix], 30 g;  *Taxillus* *chinensis* (DC.) Danser [Loranthaceae; Taxilli herba], 30 g;  *Cuscuta* *chinensis* Lam. [Convolvulaceae; Cuscutae semen], 30 g;  *Equus* *asinus* L. [Equidae; Asini corii colla], 10 g;  *Dipsacus* *asper* Wall. ex Henry [Dipsacaceae; Dipsaci radix], 15 g;  *Atractylodes* *macrocephala* Koidz. [Asteraceae; Atractylodis macrocephalae rhizoma], 10 g;  *Scutellaria* *baicalensis* Georgi [Lamiaceae; Scutellariae radix], 15 g;  *Paeonia* *lactiflora* Pall. [Ranunculaceae Juss.; Paeoniae radix alba], 25 g;  *Ligustrum* *lucidum* Ait. [Oleaceae; Ligustri lucidi fructus], 15 g;  *Eclipta* *prostrata* L. [Asteraceae; Ecliptae herba], 15 g;  *Glycyrrhiza* *uralensis* Fisch. [Fabaceae; Glycyrrhizae radix et rhizoma], 5 g;  *Rehmannia* *glutinosa* Libosch. [Scrophulariaceae; Rehmanniae radix], 15 g. | Administration: p.o.  Dose: 300 mL of water extraction  Dosing: Bid.  Duration: 14 days  Add water and decoct for 1 h. Remove the dregs and obtain 300 mL of the water decoction. |
| 21 | Xiaoping Long, 2018 | Bushen Huoxue recipe | *Cuscuta* *chinensis* Lam. [Convolvulaceae; Cuscutae semen], 15 g;  *Taxillus* *chinensis* (DC.) Danser [Loranthaceae; Taxilli herba], 10 g;  *Dipsacus* *asper* Wall. ex Henry [Dipsacaceae; Dipsaci radix], 10 g;  *Rehmannia* *glutinosa* Libosch. [Scrophulariaceae; Rehmanniae radix praeparata], 10 g;  *Atractylodes* *macrocephala* Koidz. [Asteraceae; Atractylodis macrocephalae rhizoma], 10 g;  *Boehmeria* *nivea* (L.) Gaudich. [Urticaceae; Radix boehmeriae], 15 g;  *Scutellaria* *baicalensis* Georgi [Lamiaceae; Scutellariae radix], 10 g;  *Salvia* *miltiorrhiza* Bge. [Lamiaceae; Salviae miltiorrhizae radix et rhizoma], 15 g;  *Angelica* *sinensis* (Oliv.) Diels [Apiaceae; Angelicae sinensis radix], 10 g;  *Psoralea* *corylifolia* L. [Fabaceae; Psoraleae fructus], 6 g;  *Eclipta* *prostrata* L. [Asteraceae; Ecliptae herba], 10 g;  *Agrimonia* *pilosa* Ledeb. [Rosaceae; Agrimoniae herba], 10 g. | Administration: p.o.  Dose: 300 mL of water extraction  Dosing: Bid.  Duration: 14 days  The detailed extraction procedure was not reported in the manuscript. |
| 22 | Wanzeng Ma 2019 | Guben Antai decoction | *Cuscuta chinensis* Lam. [Convolvulaceae; Cuscutae semen],15 g;  *Codonopsis pilosula* (Franch.) Nannf. [Campanulaceae; Codonopsis radix], 15 g;  *Equus asinus* L*.* [Equidae; Asini corii colla], 10 g;  *Taxillus chinensis* (DC.) Danser [Loranthaceae; Taxillus herba], 10 g;  *Dipsacus asper* Wall. ex Henry [Dipsacaceae; Dipsaci radix], 10 g;  *Atractylodes macrocephala* Koidz. [Asteraceae; Atractylodis macrocephalae rhizoma], 10 g;  *Scutellaria baicalensis* Georgi [Lamiaceae; Scutellariae radix], 10 g;  *Paeonia lactiflora* Pall. [Ranunculaceae Juss.; Paeoniae radix alba], 10 g;  *Artemisia argyi* Levl.et Vant. [Asteraceae; Artemisiae argyi folium], 6 g;  *Amomum villosum* Lour. [Zingiberaceae; Amomi fructus], 10 g. | Administration: p.o.  Dose: 200 mL of water decoction  Dosing: Bid.  Duration: 14 days  The detailed extraction procedure was not reported in the manuscript. |
| 23 | Yuru Mi 2021 | Guyuan Wentai decoction | *Astragalus membranaceus* (Fisch.) Bge.var.*mongholicus*（Bge.）Hsiao [Fabaceae; Astragali radix], 50 g;  *Paeonia lactiflora* Pall. [Ranunculaceae Juss.; Paeoniae radix alba], 30 g;  *Rehmannia glutinosa* Libosch*.*[Scrophulariaceae; Rehmanniae radix], 20 g;  *Eucommia ulmoides* Oliv. [Eucommiaceae; Eucommiae cortex], 20 g;  *Dioscorea opposita* Thunb. [Dioscoreaceae; Dioscoreae rhizoma], 20 g;  *Codonopsis pilosula* (Franch.) Nannf. [Campanulaceae; Codonopsis radix], 20 g;  *Atractylodes macrocephala* Koidz. [Asteraceae; Atractylodis macrocephalae rhizoma], 20g ;  *Cuscuta chinensis* Lam. [Convolvulaceae; Cuscutae semen], 20 g;  *Taxillus chinensis* (DC.) Danser [Loranthaceae; Taxillus herba], 20g ;  *Dipsacus asper* Wall. ex Henry [Dipsacaceae; Dipsaci radix], 20 g;  *Equus asinus* L. [Equidae; Asini corii colla], 15 g;  *Glycyrrhiza uralensis* Fisch. [Fabaceae; Glycyrrhizae radix et rhizoma], 15 g. | Administration: p.o.  Dose: water decoction  Dosing: Bid.  Duration: 14 days  The detailed extraction procedure was not reported in the manuscript. |
| 24 | Xiaxin Nong 2019 | Tanyuan yin | *Codonopsis pilosula* (Franch.) Nannf. [Campanulaceae; Codonopsis radix], 15 g;  *Taxillus chinensis* (DC.) Danser [Loranthaceae; Taxillus herba], 15 g;  *Eucommia ulmoides* Oliv. [Eucommiaceae; Eucommiae cortex], 12 g;  *Rehmannia glutinosa* Libosch*.* [Scrophulariaceae; Rehmanniae radix praeparata], 12 g;  *Atractylodes macrocephala* Koidz. [Asteraceae; Atractylodis macrocephalae rhizoma], 10 g;  *Paeonia lactiflora* Pall. [Ranunculaceae Juss.; Paeoniae radix alba], 10 g;  *Angelica sinensis* (Oliv.) Diels [Apiaceae; Angeliccae sinensis radix], 10 g;  *Citrus reticulata* Blanco [Rutaceae; Citri reticulatae pericarpium], 5 g;  *Glycyrrhiza uralensis* Fisch. [Fabaceae; Glycyrrhizae radix et rhizoma], 5 g. | Administration: p.o.  Dose: 400 mL of water decoction  Dosing: Bid.  Duration: 14 days  The detailed extraction procedure was not reported in the manuscript. |
| 25 | Xintong Shang 2021 | Gushen Antan decoction | *Codonopsis pilosula* (Franch.) Nannf. [Campanulaceae; Codonopsis radix], 15 g;  *Atractylodes macrocephala* Koidz. [Asteraceae; Atractylodis macrocephalae rhizoma], 12 g;  *Scutellaria baicalensis* Georgi [Lamiaceae; Scutellariae radix], 9 g;  *Astragalus membranaceus* (Fisch.) Bge.var.*mongholicus*(Bge.) Hsiao [Fabaceae; Astragali radix], 20 g;  *Paeonia lactiflora* Pall. [Ranunculaceae Juss.; Paeoniae radix alba], 12 g;  *Glycyrrhiza uralensis* Fisch. [Fabaceae; Glycyrrhizae radix et rhizoma], 3 g;  *Rehmannia glutinosa* Libosch. [Scrophulariaceae; Rehmanniae radix praeparata], 15 g;  *Amomum villosum* Lour. [Zingiberaceae; Amomi fructus], 5 g;  *Viscum coloratum* (Komar.) Nakai [Loranthaceae; Visci herba], 15 g. | Administration: p.o.  Dose: 400 mL of water decoction  Dosing: Bid.  Duration: 14 days  The detailed extraction procedure was not reported in the manuscript. |
| 26 | Yanxia Su 2016 | Bushen Gutai decoction | *Taxillus chinensis* (DC.) Danser [Loranthaceae; Taxillus herba], 15 g;  *Cuscuta chinensis* Lam. [Convolvulaceae; Cuscutae semen], 15 g;  *Dipsacus asper* Wall. ex Henry [Dipsacaceae; Dipsaci radix], 10 g;  *Equus asinus* L. [Equidae; Asini corii colla], 10 g;  *Atractylodes macrocephala* Koidz.[Asteraceae; Atractylodis macrocephalae rhizoma], 10 g;  *Cibotium barometz* (L.) J.Sm.[Dicksoniaceae; Cibotii rhizoma], 10 g;  *Codonopsis pilosula* (Franch.) Nannf. [Campanulaceae; Codonopsis radix], 15 g;  *Agrimonia pilosa* Ledeb. [Rosaceae; Agrimoniae herba], 10 g;  *Cornus officinalis* Sieb. et Zucc. [Cornaceae; Corni fructus], 10 g;  *Rehmannia glutinosa* Libosch. [Scrophulariaceae; Rehmanniae radix praeparata], 10 g;  *Poria cocos* (Schw.) Wolf [Polyporaceae; poria], 15 g;  *Rosa laevigata* Michx. [Rosaceae; Rosae laevigatae fructus], 10 g;  *Perilla frutescens* (L.) Britton. [Labiatae; Perillae caulis], 10 g. | For Bushen Gutai decoction:  Administration: p.o.  Dose: 400 mL of water decoction  Dosing: Bid.  Duration: 7 days  Wash all herbs; add pure water to cover the surface of the herbs for 2 cm to 3 cm; decoct for 30 min; separate the liquid; add pure water to the dregs and decoct for 1 h; separate the liquid; combine the two liquids to 400 mL.  For Antai powder navel:  Mash and take 5 g to apply the umbilicus; and remove the medicine after the fetus is safe. |
| 27 | Lidan Teng2018 | Xuanyu Tongjing decoction | *Paeonia lactiflora* Pall. [Ranunculaceae Juss.; Paeoniae radix alba], 10 g;  *Angelica sinensis* (Oliv.) Diels [Apiaceae; Angeliccae sinensis radix], 10 g;  *Paeonia suffruticosa* Andr. [Ranunculaceae; Moutan cortex], 6 g;  *Gardenia jasminoides* Ellis [Rubiaceae Juss.; Cape jasmine fruit], 10 g;  *Sinapis alba* L. [Brassicaceae; Semen sinapis albae], 10 g;  *Bupleurum chinense* DC. [Umbelliferae; Bupleuri radix], 6 g;  *Cyperus rotundus* L. [Cyperaceae; Cyperi rhizoma], 10 g;  *Curcuma longa* L. [Zingiberaceae; Curcuma sichuanensisx. x. chen], 10 g;  *Scutellaria baicalensis* Georgi [Lamiaceae; Scutellariae radix], 10 g;  *Glycyrrhiza uralensis* Fisch. [Fabaceae; Glycyrrhizae radix et rhizoma], 3 g. | Administration: p.o.  Dose: 400 mL of water decoction  Dosing: Bid.  Duration: 30 days  The detailed extraction procedure was not reported in the manuscript. |
| 28 | Yuanmei Wang 2019 | Bushen Antai decoction | *Dipsacus asper* Wall. ex Henry [Dipsacaceae; Dipsaci radix], 15 g;  *Taxillus chinensis* (DC.) Danser [Loranthaceae; Taxillus herba], 15 g;  *Cuscuta chinensis* Lam. [Convolvulaceae; Cuscutae semen], 15 g;  *Eucommia ulmoides* Oliv. [Eucommiaceae; Eucommiae cortex], 15 g;  *Atractylodes macrocephala* Koidz. [Asteraceae; Atractylodis macrocephalae rhizoma], 15 g;  *Codonopsis pilosula* (Franch.) Nannf. [Campanulaceae; Codonopsis radix], 15 g;  *Boehmeria nivea* (L.) Gaudich. [ Urticaceae; Radix boehmeriae], 15 g;  *Equus asinus* L. [Equidae; Asini corii colla], 10 g;  *Scutellaria baicalensis* Georgi [Lamiaceae; Scutellariae radix], 8 g;  *Perilla frutescens* (L.) Britton. [Labiatae; Perillae caulis], 8 g;  *Amomum villosum* Lour. [Zingiberaceae; Amomi fructus], 5 g. | Administration: p.o.  Dose: 300 mL of water decoction  Dosing: Bid.  Duration: 10-14 days  The detailed extraction procedure was not reported in the manuscript. |
| 29 | Chaoxia Xiao; 2008 | Buqi Yangxue Gutai decoction | *Cuscuta chinensis* Lam. [Convolvulaceae; Cuscutae semen], 10 g;  *Taxillus chinensis* (DC.) Danser [Loranthaceae; Taxilli herba], 15 g;  *Dipsacus asper* Wall. ex Henry [Dipsacaceae; Dipsaci radix], 10 g;  *Eucommia ulmoides* Oliv. [Eucommiaceae; Eucommiae cortex], 15 g;  *Astragalus membranaceus* (Fisch.) Bge.var.*mongholicus* (Bge.) Hsiao [Fabaceae; Astragali radix], 20 g;  *Panax ginseng C*. A. Mey. [Araliaceae; Ginseng radix et rhizoma], 10 g;  *Dioscorea opposita* Thunb. [Dioscoreaceae; Dioscoreae rhizoma], 15 g;  *Paeonia lactiflora* Pall. [Ranunculaceae Juss.; Paeoniae radix alba], 15 g;  *Equus asinus* L. [Equidae; Asini corii colla], 15 g;  *Rehmannia glutinosa* Libosch. [Scrophulariaceae; Rehmanniae radix praeparata], 10 g;  *Citrus reticulata* Blanco [Rutaceae; Citri reticulatae pericarpium], 5 g | Administration: p.o.  Dose: Water extraction  Dosing: Bid.  Duration: 5-7 days  The detailed extraction procedure was not reported in manuscript. |
| 30 | Na Xin 2018 | Zishen Yutai pill | *Cuscuta chinensis* Lam. [Convolvulaceae; Cuscutae semen];  *Amomum villosum* Lour. [Zingiberaceae; Amomi fructus];  *Rehmannia glutinosa* Libosch. [Scrophulariaceae; Rehmanniae radix praeparata];  *Panax ginseng* C.A.Mey. [Araliaceae; Ginseng radix et rhizoma];  *Taxillus chinensis* (DC.) Danser [Loranthaceae; Taxillus herba];  Equus asinus L. [Equidae; Asini corii colla];  *Pleuropterus multiflorus* (Thunb.) Nakai [Polygonaceae; Polygoni multiflori radix];  *Artemisia argyi* Levl.et Vant. [Asteraceae; Artemisiae argyi folium];  *Morinda officinalis* How [Rubiaceae; Morindae officinalis radix];  *Atractylodes macrocephala* Koidz.[Asteraceae; Atractylodis macrocephalae rhizoma];  *Codonopsis pilosula* (Franch.) Nannf. [Campanulaceae; Codonopsis radix];  *Cervus elaphus* Linnaeus [Cervidae; Cervi Cornu Degelatinatum];  *Dipsacus asper* Wall. ex Henry [Dipsacaceae; Dipsaci radix];  *Eucommia ulmoides* Oliv. [Eucommiaceae; Eucommiae cortex].  The amount of each drug in a polyherbal preparation is unavailable in original text. | Zishen Yutai Pill (SFDA approval No.: Z44020008)  Guangzhou Baiyunshan Zhongyi Pharmaceutical Co.  Administration: p.o.  Dose: 5 g of pills  Dosing: Tid.  Duration: 14 days  The detailed extraction procedure has not been addressed in manuscript. |
| 31 | Mingqun Yang 2006 | Chinese herbal medicine | *Equus asinus* L. [Equidae; Asini corii colla], 15 g;  *Artemisia argyi* Levl.et Vant. [Asteraceae; Artemisiae argyi folium], 12 g;  *Rehmannia glutinosa* Libosch. [Scrophulariaceae; Rehmanniae radix praeparata], 20 g;  *Ligusticum chuanxiong* Hort. [Apiaceae; Chuanxiong rhizoma], 5 g;  *Paeonia lactiflora* Pall. [Ranunculaceae Juss.; Paeoniae radix alba], 10 g;  *Angelica sinensis* (Oliv.) Diels [Apiaceae; Angelicae sinensis radix], 10 g;  *Glycyrrhiza uralensis* Fisch. [Fabaceae; Glycyrrhizae radix et rhizoma], 6 g. | Administration: p.o.  Dose: Water extraction  Dosing: Tid.  Duration: 7-14 days  The detailed extraction procedure was not reported in manuscript. |
| 32 | Shuang Ye 2021 | Yangxue Guchong decoction | *Rehmannia glutinosa* Libosch. [Scrophulariaceae; Rehmanniae radix praeparata], 12 g;  *Dipsacus asper* Wall. ex Henry [Dipsacaceae; Dipsaci radix], 12 g;  *Taxillus chinensis* (DC.) Danser [Loranthaceae; Taxillus herba], 12 g;  *Equus asinus* L. [Equidae; Asini corii colla], 12 g;  *Cuscuta chinensis* Lam. [Convolvulaceae; Cuscutae semen], 20 g;  *Angelica sinensis* (Oliv.) Diels [Apiaceae; Angeliccae sinensis radix], 9 g;  *Paeonia lactiflora* Pall. [Ranunculaceae Juss.; Paeoniae radix alba], 9 g;  Artemisia argyi Levl.et Vant. [Asteraceae; Artemisiae argyi folium], 9 g;  *Ligusticum chuanxiong* Hort. [Apiaceae; Chuanxiong rhizoma], 6 g;  *Glycyrrhiza uralensis* Fisch. [Fabaceae; Glycyrrhizae radix et rhizoma], 6 g. | Administration: p.o.  Dose: 300 mL of water decoction  Dosing: Tid.  Duration: 14 days  The detailed extraction procedure was not reported in the manuscript. |
| 33 | Jia Yu 2019 | Traditional Chinese medicine formula granules | *Angelica sinensis* (Oliv.) Diels [Apiaceae; Angeliccae sinensis radix], 10 g;  *Paeonia lactiflora* Pall. [Ranunculaceae Juss.; Paeoniae radix rubra], 10 g;  *Panax notoginseng* (Burk.) F. H. Chen [Araliaceae; Notoginseng radix et rhizoma], 3 g;  *Taxillus chinensis* (DC.) Danser [Loranthaceae; Taxillus herba], 15 g;  *Dipsacus asper* Wall. ex Henry [Dipsacaceae; Dipsaci radix], 15 g;  *Cuscuta chinensis* Lam. [Convolvulaceae; Cuscutae semen], 20 g;  *Atractylodes macrocephala* Koidz. [Asteraceae; Atractylodis macrocephalae rhizoma], 12 g;  *Astragalus membranaceus* (Fisch.) Bge.var.*mongholicus* (Bge.) Hsiao [Fabaceae; Astragali radix], 20 g;  *Glycyrrhiza uralensis* Fisch. [Fabaceae; Glycyrrhizae radix et rhizoma], 3 g. | Administration: p.o.  Dose: water decoction  Dosing: Bid.  Duration: 21 days  The detailed extraction procedure was not reported in the manuscript. |
| 34 | Lin Yu 2021 | Zishen Yutai pill | *Cuscuta chinensis* Lam. [Convolvulaceae; Cuscutae semen];  *Amomum villosum* Lour. [Zingiberaceae; Amomi fructus];  *Rehmannia glutinosa* Libosch. DC. [Scrophulariaceae; Rehmanniae radix praeparata];  *Panax ginseng* C.A.Mey. [Araliaceae; Ginseng radix et rhizoma];  *Taxillus chinensis* (DC.) Danser [Loranthaceae; Taxillus herba];  Equus asinus L. [Equidae; Asini corii colla];  *Pleuropterus multiflorus* (Thunb.) Nakai [Polygonaceae; Polygoni multiflori radix];  *Artemisia argyi* Levl.et Vant. [Asteraceae; Artemisiae argyi folium];  *Morinda officinalis* How [Rubiaceae; Morindae officinalis radix];  *Atractylodes macrocephala* Koidz.[Asteraceae; Atractylodis macrocephalae rhizoma];  *Codonopsis pilosula* (Franch.) Nannf. [Campanulaceae; Codonopsis radix];  *Cervus elaphus* Linnaeus [Cervidae; Cervi cornu degelatinatum];  *Dipsacus asper* Wall. ex Henry [Dipsacaceae; Dipsaci radix];  *Eucommia ulmoides* Oliv. [Eucommiaceae; Eucommiae cortex].  The amount of each drug in a polyherbal preparation is unavailable in original text. | Zishen Yutai pills (SFDA approval No.: Z44020008)  Guangzhou Baiyunshan Zhongyi Pharmaceutical Co..  Administration: p.o.  Dose: 5 g of pills  Dosing: Tid.  Duration: 21 days  The detailed extraction procedure was not reported in manuscript. |
| 35 | Xinhui Yu 2021 | Bushen Antai decoction | *Cuscuta chinensis* Lam. [Convolvulaceae; Cuscutae semen];  *Dipsacus asper* Wall. ex Henry [Dipsacaceae; Dipsaci radix];  *Taxillus chinensis* (DC.) Danser [Loranthaceae; Taxillus herba];  *Rehmannia glutinosa* Libosch*.* [Scrophulariaceae; Rehmanniae radix praeparata];  *Astragalus membranaceus* (Fisch.) Bge.var.*mongholicus* (Bge.) Hsiao [Fabaceae; Astragali radix];  *Atractylodes macrocephala* Koidz. [Asteraceae; Atractylodis macrocephalae rhizoma];  *Codonopsis pilosula* (Franch.) Nannf. [Campanulaceae; Codonopsis radix];  *Boehmeria nivea* (L.) Gaudich. [Urticaceae; Radix boehmeriae].  The amount of each drug in a polyherbal preparation is unavailable in original text. | Bushen Antai Decoction (SFDA approval No.: BZ20080017)  Administration: p.o.  Dose: 120 mL of water decoction  Dosing: Tid.  Duration: 14 days |
| 36 | Xinming Zhang 2008 | Shoutai pill | *Cuscuta chinensis* Lam. [Convolvulaceae; Cuscutae semen], 20 g;  *Taxillus chinensis* (DC.) Danser [Loranthaceae; Taxilli herba], 25 g;  *Equus asinus* L. [Equidae; Asini corii colla], 15 g;  *Dipsacus asper* Wall. ex Henry [Dipsacaceae; Dipsaci radix], 15 g;  *Rehmannia glutinosa* Libosch. [Scrophulariaceae; Rehmanniae radix praeparata], 15 g;  *Codonopsis pilosula* (Franch.) Nannf. [Campanulaceae; Codonopsis radix], 15 g;  *Atractylodes macrocephala* Koidz. [Asteraceae; Atractylodis macrocephalae rhizoma], 15 g;  *Glycyrrhiza uralensis* Fisch. [Fabaceae; Glycyrrhizae radix et rhizoma], 10 g. | Administration: p.o.  Dose: Water extraction  Dosing: Bid.  Duration: 7 days  The detailed extraction procedure was not reported in manuscript. |
| 37 | Wei Zhang 2015 | Yishen Angong recepie | For patients with symptoms of renal deficiency and blood fever:  *Rehmannia glutinosa* Libosch. [Scrophulariaceae; Rehmanniae radix], 15 g;  *Rehmannia glutinosa* Libosch*.* [Scrophulariaceae; Rehmanniae radix praeparata], 15 g;  *Paeonia lactiflora* Pall. [Ranunculaceae Juss.; Paeoniae radix alba], 15 g;  *Dioscorea opposita* Thunb. [Dioscoreaceae; Dioscoreae rhizoma], 15 g;  *Dipsacus asper* Wall. ex Henry [Dipsacaceae; Dipsaci radix], 15 g;  *Scutellaria baicalensis* Georgi [Lamiaceae; Scutellariae radix], 15 g;  *Taxillus chinensis* (DC.) Danser [Loranthaceae; Taxillus herba], 15 g;  *Equus asinus* L. [Equidae; Asini corii colla], 12 g;  *Eclipta prostrata* L. [Compositae; Ecliptae herba], 12 g;  *Glycyrrhiza uralensis* Fisch. [Fabaceae; Glycyrrhizae radix et rhizoma], 5 g.  For patients with symptoms of spleen and kidney deficiency:  *Codonopsis pilosula* (Franch.) Nannf. [Campanulaceae; Codonopsis radix], 15 g;  *Atractylodes macrocephala* Koidz. [Asteraceae; Atractylodis macrocephalae rhizoma], 12 g;  *Dipsacus asper* Wall. ex Henry [Dipsacaceae; Dipsaci radix], 12 g;  *Taxillus chinensis* (DC.) Danser [Loranthaceae; Taxillus herba], 15 g;  *Eucommia ulmoides* Oliv. [Eucommiaceae; Eucommiae cortex], 15 g;  *Cuscuta chinensis* Lam. [Convolvulaceae; Cuscutae semen], 30 g;  *Rehmannia glutinosa* Libosch. [Scrophulariaceae; Rehmanniae radix praeparata], 15 g;  *Poria cocos* (Schw.) Wolf [Polyporaceae; Poria], 12 g;  *Equus asinus* L. [Equidae; Asini corii colla], 12 g;  *Glycyrrhiza uralensis* Fisch. [Fabaceae; Glycyrrhizae radix et rhizoma], 5 g. | Administration: p.o.  Dose: water decoction  Dosing: Bid.  Duration: 21 days  The detailed extraction procedure was not reported in the manuscript. |
| 38 | Dongming Zhang 2017 | Ershan Yangshi Baotai decoction | *Astragalus membranaceus* (Fisch.) Bge.var. *mongholicus* (Bge.) Hsiao [Fabaceae; Astragali radix], 20 g;  *Ligustrum lucidum* Ait. [Oleaceae; Ligustri lucidi fructus], 20 g;  *Paeonia lactiflora* Pall. [Ranunculaceae Juss.; Paeoniae radix alba], 15 g;  *Cuscuta chinensis* Lam. [Convolvulaceae; Cuscutae semen], 15 g;  *Dioscorea opposita* Thunb. [Dioscoreaceae; Dioscoreae rhizoma], 15 g;  *Eclipta prostrata* L. [Compositae; Ecliptae herba], 15 g;  *Eucommia ulmoides* Oliv. [Eucommiaceae; Eucommiae cortex], 15 g;  *Rehmannia glutinosa* Libosch. [Scrophulariaceae; Rehmanniae radix], 12 g;  *Rehmannia glutinosa* Libosch. [Scrophulariaceae; Rehmanniae radix praeparata], 12 g;  *Paeonia suffruticosa* Andr. [Ranunculaceae; Moutan cortex], 12 g;  *Taxillus chinensis* (DC.) Danser [Loranthaceae; Taxillus herba], 12 g;  *Scutellaria baicalensis* Georgi [Lamiaceae; Scutellariae radix], 12 g;  *Poria cocos* (Schw.) Wolf [Polyporaceae; Poria], 12 g;  *Boehmeria nivea* (L.) Gaudich. [Urticaceae; Radix boehmeriae], 12 g;  *Dipsacus asper* Wall. ex Henry [Dipsacaceae; Dipsaci radix], 10 g. | Administration: p.o.  Dose: 400 mL of water decoction  Dosing: Bid.  Duration: 21 days  The detailed extraction procedure was not reported in the manuscript. |
| 39 | Zhirong Zhang 2019 | Bushen Jianpi decoction | *Taxillus chinensis* (DC.) Danser [Loranthaceae; Taxilli herba], 30 g;  *Eucommia ulmoides* Oliv. [Eucommiaceae; Eucommiae cortex]. 30 g;  *Dipsacus asper* Wall. ex Henry [Dipsacaceae; Dipsaci radix], 30 g;  *Lycium barbarum* L. [Solanaceae; Lycii fructus], 20 g;  *Polygala tenuifolia* Willd. [Polygalaceae; Polygalae radix], 15 g;  *Homo sapiens* [Hominidae; Crinis carbonisatus], 15 g;  *Codonopsis pilosula* (Franch.) Nannf. [Campanulaceae; Codonopsis radix], 10 g;  *Cuscuta chinensis* Lam. [Convolvulaceae; Cuscutae semen], 10 g;  *Panax ginseng* C.A.Mey. [Araliaceae; Ginseng radix et rhizoma rubra], 10 g;  *Glycyrrhiza uralensis* Fisch. [Fabaceae; glycyrrhizae radix et rhizoma], 10 g;  *Atractylodes macrocephala* Koidz. [Asteraceae; Atractylodis macrocephalae rhizoma], 10 g;  *Scutellaria baicalensis* Georgi [Lamiaceae; Scutellariae radix], 10 g;  *Amomum villosum* Lour. [Zingiberaceae; Amomi fructus], 10 g;  *Cimicifuga heracleifolia* Kom. [Ranunculaceae; Cimicifugae rhizoma], 10 g. | Administration: p.o.  Dose: 400 mL of water extraction  Dosing: Bid.  Duration: 20 days  The detailed extraction procedure was not reported in manuscript. |
| 40 | Huanzhen Zhang 2020 | Bushen Baotai Zhuyun decoction | *Cuscuta chinensis* Lam. [Convolvulaceae; Cuscutae semen], 20 g;  *Atractylodes macrocephala* Koidz. [Asteraceae; Atractylodis macrocephalae rhizoma], 15 g;  *Taxillus chinensis* (DC.) Danser [Loranthaceae; Taxillus herba], 15 g;  *Codonopsis pilosula* (Franch.) Nannf. [Campanulaceae; Codonopsis radix], 15 g;  *Astragalus membranaceus* (Fisch.) Bge.var.*mongholicus*(Bge.) Hsiao [Fabaceae; Astragali radix], 15 g;  *Dioscorea opposita* Thunb. [Dioscoreaceae; Dioscoreae rhizoma], 15 g;  *Dipsacus asper* Wall. ex Henry [Dipsacaceae; Dipsaci radix], 15 g;  *Eucommia ulmoides* Oliv. [Eucommiaceae; Eucommiae cortex], 15 g;  *Boehmeria nivea* (L.) Gaudich. [Urticaceae; Radix boehmeriae], 12 g;  *Paeonia lactiflora* Pall. [Ranunculaceae Juss.; Paeoniae radix alba], 10 g;  *Rehmannia glutinosa* Libosch. [Scrophulariaceae; Rehmanniae radix praeparata], 10 g;  *Equus asinus* L. [Equidae; Asini corii colla], 10 g; | Administration: p.o.  Dose: 400 mL of water decoction  Dosing: Bid.  Duration: 21 days  The detailed extraction procedure was not reported in the manuscript. |
| 41 | Liping Zhang 2020 | Yuyin decoction | *Rehmannia glutinosa* Libosch. [Scrophulariaceae; Rehmanniae radix praeparata], 20 g;  *Paeonia lactiflora* Pall. [Ranunculaceae Juss.; Paeoniae radix alba], 15 g;  *Dipsacus asper* Wall. ex Henry [Dipsacaceae; Dipsaci radix], 20 g;  *Dioscorea opposita* Thunb. [Dioscoreaceae; Dioscoreae rhizoma], 20 g;  *Equus asinus* L. [Equidae; Asini corii colla], 15 g;  *Taxillus chinensis* (DC.) Danser [Loranthaceae; Taxillus herba], 20 g;  *Cornus officinalis* Sieb. et Zucc. [Cornaceae; Corni fructus], 20 g;  *SepielLa maindroni* *de* Rochebrune [Sepiidae; Sepiae endoconcha], 15 g;  *Chinemys reevesii* (Gray) [Emydidae; Testudinis carapax et plastrum], 15 g;  *Cuscuta chinensis* Lam. [Convolvulaceae; Cuscutae semen], 20 g;  *Ostrea gigas* Thunberg [Ostreidae; Ostreae concha], 15 g. | Administration: p.o.  Dose: 400 mL of water decoction  Dosing: Bid.  Duration: 20 days  The detailed extraction procedure was not reported in the manuscript. |
| 42 | Lili Zhang 2020 | Baoyin decoction | *Rehmannia glutinosa* Libosch. [Scrophulariaceae; Rehmanniae radix], 10 g;  *Rehmannia glutinosa* Libosch. [Scrophulariaceae; Rehmanniae radix praeparata], 10 g;  *Paeonia lactiflora* Pall. [Ranunculaceae Juss; Paeoniae radix alba], 10 g;  *Dipsacus asper* Wall. ex Henry [Dipsacaceae; Dipsaci radix], 10 g;  *Dioscorea opposita* Thunb. [Dioscoreaceae; Dioscoreae rhizoma], 10 g;  *Scutellaria baicalensis* Georgi [Lamiaceae; Scutellariae radix], 10 g;  *Phellodendron chinense* Schneid. [Cupressaceae; Phellodendri chinensis cortex], 6 g;  *Glycyrrhiza uralensis* Fisch. [Fabaceae; Glycyrrhizae radix et rhizoma], 3 g. | Administration: p.o.  Dose: water decoction  Dosing: Bid.  Duration: 14 days  The detailed extraction procedure was not reported in the manuscript. |
| 43 | Xue Zhang; 2021 | Shoutai pill | *Equus asinus* L. [Equidae; Asini corii colla], 9 g;*Codonopsis pilosula* (Franch.) Nannf. [Campanulaceae; Codonopsis radix], 12 g;*Atractylodes macrocephala* Koidz. [Asteraceae; Atractylodis macrocephalae rhizoma], 12 g;*Eucommia ulmoides* Oliv. [Eucommiaceae; Eucommiae cortex], 12 g;*Dipsacus asper* Wall. ex Henry [Dipsacaceae; Dipsaci radix], 12 g *Taxillus chinensis* (DC.) Danser [Loranthaceae; Taxilli herba], 18 g. | Administration: p.o.  Dose: 200 mL of water extraction  Dosing: Bid.  Duration: 14 days  The detailed extraction procedure was not reported in manuscript. |
| 44 | Huijun Zheng 2020 | Bushen Yiqi and Guchong Antai decoction | *Cuscuta chinensis* Lam. [Convolvulaceae; Cuscutae semen], 10 g;  *Eucommia ulmoides* Oliv. [Eucommiaceae; Eucommiae cortex], 10 g;  *Taxillus chinensis* (DC.) Danser [Loranthaceae; Taxilli herba], 10 g;  *Dipsacus asper* Wall. ex Henry [Dipsacaceae; Dipsaci radix], 10 g;  *Equus asinus* L. [Equidae; Asini corii colla], 10 g;  *Atractylodes macrocephala* Koidz. [Asteraceae; Atractylodis macrocephalae rhizoma], 10 g;  *Pseudostellaria heterophylla* (Miq.) Pax ex Pax et Hoffm. [Caryophyllaceae; Pseudostellariae radix], 10 g;  *Glycyrrhiza uralensis* Fisch. [Fabaceae; Glycyrrhizae radix et rhizoma], 6 g | Administration: p.o.  Dose: 200 mL of water extraction  Dosing: Bid.  Duration: 14 days  The detailed extraction procedure was not reported in manuscript. |
| 45 | Guangxia Zhu 2020 | Bushen Baotai Zhuyun decoction | *Boehmeria nivea* (L.) Gaudich. [Urticaceae; Radix boehmeriae], 30 g;  *Cuscuta chinensis* Lam. [Convolvulaceae; Cuscutae semen], 20 g;  *Taxillus chinensis* (DC.) Danser [Loranthaceae; Taxilli herba], 15 g;  *Codonopsis pilosula* (Franch.) Nannf. [Campanulaceae; Codonopsis radix], 15 g;  *Atractylodes macrocephala* Koidz. [Asteraceae; Atractylodis macrocephalae rhizoma], 15 g;  *Astragalus membranaceus* (Fisch.) Bge.var. *mongholicus* (Bge.) Hsiao [Fabaceae; Astragali radix], 15 g;  *Eucommia ulmoides* Oliv. [Eucommiaceae; Eucommiae cortex], 15 g;  *Dioscorea opposita* Thunb. [Dioscoreaceae; Dioscoreae rhizoma], 15 g;  *Dipsacus asper* Wall. ex Henry [Dipsacaceae; Dipsaci radix], 15 g;  *Paeonia lactiflora* Pall. [Ranunculaceae Juss.; Paeoniae radix alba], 10 g;  *Lycium barbarum* L. [Solanaceae; Lycii fructus], 10 g;  *Rehmannia glutinosa* Libosch. [Scrophulariaceae; Rehmanniae radix praeparata], 10 g;  *Equus asinus* L. [Equidae; Asini corii colla], 10 g  *Salvia miltiorrhiza* Bge. [Lamiaceae; Salviae miltiorrhizae radix et rhizoma], 6 g. | Administration: p.o.  Dose: 400 mL of water extraction  Dosing: Bid.  Duration: 14 days  The detailed extraction procedure was not reported in manuscript. |
| 46 | Huiqin Zhuang 2016 | Yishen Angong recipe | *Rehmannia glutinosa* Libosch. [Scrophulariaceae; Rehmanniae radix], 15 g;  *Paeonia lactiflora* Pall. [Ranunculaceae Juss.; Paeoniae radix alba], 15 g;  *Codonopsis pilosula* (Franch.) Nannf. [Campanulaceae; Codonopsis radix], 15 g;  *Rehmannia glutinosa* Libosch. [Scrophulariaceae; Rehmanniae radix praeparata], 15 g;  Dioscorea opposita Thunb. [Dioscoreaceae; Dioscoreae rhizoma], 15 g;  *Dipsacus asper* Wall. ex Henry [Dipsacaceae; Dipsaci radix], 15 g;  *Taxillus chinensis* (DC.) Danser [Loranthaceae; Taxilli herba], 15 g;  *Eucommia ulmoides* Oliv. [Eucommiaceae; Eucommiae cortex], 15 g;  *Scutellaria baicalensis* Georgi [Lamiaceae; Scutellariae radix], 15 g;  *Cuscuta chinensis* Lam. [Convolvulaceae; Cuscutae semen], 30 g;  *Eclipta prostrata* L. [Asteraceae; Ecliptae herba], 12 g;  *Equus asinus* L. [Equidae; Asini Corii colla], 12 g;  *Poria cocos* (Schw.) Wolf [Polyporaceae; Poria], 12 g;  *Atractylodes macrocephala* Koidz. [Asteraceae; Atractylodis macrocephalae rhizoma], 12 g;  *Glycyrrhiza uralensis* Fisch. [Fabaceae; Glycyrrhizae radix et rhizoma], 5 g. | Administration: p.o.  Dose: 300 mL of water extraction  Dosing: Tid.  Duration: 21 days  Add 300 mL of water; boil and keep it for 15min to obtain the water decoction. |

Abbreviations: Q.d: once per day (quaque die); Bid: twice per day (bis in die); Tid: three times per day (ter in die); p.o.: oral administration (per os.).

All taxonomical information was extracted and validated from the Chinese Pharmacopoeia 2020 version.

| **CHM alone versus WM alone.** | | | | |
| --- | --- | --- | --- | --- |
| **No.** | **Study ID** | **Prescription/**  **(Chinese formulae)** | **Raw material of botanical drugs** | **Preparation or details** |
| 1 | Zuzhen Duan 2016 | Bushen Jianpi Antai decoction | *Cuscuta chinensis* Lam. [Convolvulaceae; Cuscutae semen], 30g;  *Taxillus chinensis* (DC.) Danser [Loranthaceae; Taxilli herba], 15g;  *Dipsacus asper* Wall*.* ex Henry [Dipsacaceae; Dipsaci radix], 10g;  *Equus asinus* L. [Equidae; Asini Corii Colla], 10g;  *Dioscorea opposita* Thunb. [Dioscoreaceae; Dioscoreae rhizoma], 20g;  *Codonopsis pilosula* (Franch.) Nannf. [Campanulaceae; Codonopsis radix], 15g;  *Atractylodes macrocephala* Koidz. [Asteraceae; Atractylodis macrocephalae rhizome], 15g;  *Perilla frutescens* (L.) Britton [Lamiaceae; Perillae caulis], 10g;  *Cornus officinalis* Sieb. et Zucc. [Cornaceae; Corni fructus], 15g;  *Lycium barbarum* L. [Solanaceae; Lycii fructus], 15g;  *Glycyrrhiza uralensis* Fisch. [Fabaceae; Glycyrrhizae radix et rhizoma], 5g. | Administration: p.o.  Dose: 150 mL of water extraction  Dosing: Bid.  Duration: 14 days  The detailed extraction procedure was not reported in the manuscript. |
| 2 | Guixiu Jiang 2019 | Chinese medicine recipe | 1. For patients with severe lower back pain:   *Citrus reticulata* Blanco [Rutaceae; Citri reticulatae pericarpium], 9g;  *Glycyrrhiza uralensis* Fisch. [Fabaceae; Glycyrrhizae radix et rhizoma], 10g;  *Rehmannia glutinosa* Libosch. [Scrophulariaceae; Rehmanniae radix praeparata], 10g;  *Atractylodes macrocephala* Koidz. [Asteraceae; Atractylodis macrocephalae rhizoma], 15g  *Dipsacus asper* Wall. ex Henry [Dipsacaceae, Dipsaci radix], 15g.   1. Patients with symptoms of Yin deficiency and internal heat:   *Phellodendron chinense Schneid.* [Cupressaceae; Phellodendri chinensis cortex], 10g;  Rehmannia glutinosa Libosch. [Scrophulariaceae; Rehmanniae radix praeparata], 10g; | Administration: p.o.  Dose: 500 mL of water extraction  Dosing: Bid.  Duration: 14 days  The detailed extraction procedure was not reported in manuscript. |

|  |  |  | *Scutellaria baicalensis* Georgi [Lamiaceae; Scutellariae radix], 10g;  *Paeonia lactiflora* Pall. [Ranunculaceae Juss.; Paeoniae radix alba], 15g;  *Dioscorea opposita* Thunb. [Dioscoreaceae; Dioscoreae rhizoma], 15g;  *Dipsacus asper* Wall. ex Henry [Dipsacaceae; Dipsaci radix], 15g  *Dioscorea opposita* Thunb. [Dioscoreaceae; Dioscoreae rhizoma], 15g.   1. Patients with symptoms of spleen and kidney deficiency:   *Atractylodes macrocephala* Koidz. [Asteraceae; Atractylodis macrocephalae rhizoma], 9g;  *Taxillus chinensis* (DC.) Danser [Loranthaceae; Taxilli herba], 9g;  *Equus asinus* L. [Equidae; Asini corii colla], 10g;  *Codonopsis pilosula* (Franch.) Nannf. [Campanulaceae; Codonopsis radix], 15g;  *Dipsacus asper* Wall. ex Henry [Dipsacaceae; Dipsaci radix], 15g  *Cuscuta chinensis* Lam. [Convolvulaceae; Cuscutae semen], 20g.  4] Trauma type patients:  *Angelica sinensis* (Oliv.) Diels [Apiaceae; Angelicae sinensis radix], 6g;  *Rehmannia glutinosa* Libosch. [Scrophulariaceae; Rehmanniae radix praeparata], 10g;  *Paeonia lactiflora* Pall. [Ranunculaceae Juss.; Paeoniae radix alba], 10g;  *Astragalus membranaceus* (Fisch.) Bge.var.*mongholicus* (Bge.) Hsiao [Fabaceae; Astragali radix], 10g;  *Taxillus chinensis* (DC.) Danser [Loranthaceae; Taxilli herba], 10g;  *Codonopsis pilosula* (Franch.) Nannf. [Campanulaceae; Codonopsis radix], 10g;  *Dipsacus asper* Wall. ex Henry [Dipsacaceae; Dipsaci radix], 15g;  *Cuscuta chinensis* Lam. [Convolvulaceae; Cuscutae semen], 15g. |  |
| --- | --- | --- | --- | --- |
| 3 | Yanqin Li 2020 | Jiawei Shoutai pill | *Dipsacus asper* Wall. ex Henry [Dipsacaceae; Dipsaci radix], 12g;  *Eucommia ulmoides* Oliv. [Eucommiaceae; Eucommiae cortex], 12g;  *Cuscuta chinensis* Lam. [Convolvulaceae; Cuscutae semen], 9g;  *Codonopsis pilosula* (Franch.) Nannf. [Campanulaceae; Codonopsis radix], 9g;  *Atractylodes macrocephala* Koidz. [Asteraceae; Atractylodis macrocephalae rhizoma], 9g;  *Scutellaria baicalensis* Georgi [Lamiaceae; Scutellariae radix], 6g;  *Equus asinus* L. [Equidae; Asini corii colla], 6g;  *Paeonia lactiflora* Pall. [Ranunculaceae Juss.; Paeoniae radix alba], 6g;  *Amomum villosum* Lour. [Zingiberaceae; Amomi fructus], 3g;  *Perilla frutescens* (L.) Britton [Lamiaceae; Perillae caulis], 6g;  *Eclipta prostrata* L. [Asteraceae; Ecliptae herba], 3g. | Administration: p.o.  Dose: 200 mL of water extraction  Dosing: Q.d.  Duration: 14 days  The detailed extraction procedure was not reported in manuscript. |
| 4 | Zhihui Liu 2012 | Antai decoction | *Taxillus chinensis* (DC.) Danser [Loranthaceae; Taxilli herba], 20g;  *Cuscuta chinensis* Lam. [Convolvulaceae; Cuscutae semen], 15g;  *Dipsacus asper* Wall. ex Henry [Dipsacaceae; Dipsaci radix], 15g;  *Equus asinus* L. [Equidae; Asini Corii Colla], 10g;  *Codonopsis pilosula* (Franch.) Nannf. [Campanulaceae; Codonopsis radix], 20g;  *Astragalus membranaceus* (Fisch.) Bge.var.*mongholicus* (Bge.) Hsiao [Fabaceae; Astragali radix], 15g;  *Atractylodes macrocephala* Koidz. [Asteraceae; Atractylodis macrocephalae rhizoma], 10g;  *Dioscorea opposita* Thunb. [Dioscoreaceae，Dioscoreae rhizoma], 15g;  *Perilla frutescens* (L.) Britton [Lamiaceae; Perillae caulis], 10g;  *Boehmeria nivea* (L.) Gaudich. [Urticaceae; Radix boehmeriae], 15g;  *Glycyrrhiza uralensis* Fisch. [Fabaceae; Glycyrrhizae radix et rhizoma], 10g | Administration: p.o.  Dose: Water extraction  Dosing: Bid.  Duration: 14 days  The detailed extraction procedure was not reported in manuscript. |
| 5 | Yanling Luo 2020 | Bushen Yiqi Antai decoction | *Codonopsis pilosula* (Franch.) Nannf. [Campanulaceae; Codonopsis radix], 10g;  *Atractylodes macrocephala* Koidz. [Asteraceae; Atractylodis macrocephalae rhizoma], 10g;  *Poria cocos* (Schw.) Wolf [Polyporaceae; Poria], 10g;  *Scutellaria baicalensis* Georgi [Lamiaceae; Scutellariae radix], 10g;  *Paeonia lactiflora* Pall. [Ranunculaceae Juss.; Paeoniae radix alba], 10g;  *Cyperus rotundus* L. [Cyperaceae, Cyperi rhizoma], 10g;  *Artemisia argyi* Levl.et Vant. [Asteraceae; Artemisiae argyi folium], 10g;  *Angelica sinensis* (Oliv.) Diels [Apiaceae; Angelicae sinensis radix], 10g;  *Rehmannia glutinosa* Libosch. [Scrophulariaceae; Rehmanniae radix praeparata], 10g. | Administration: p.o.  Dose: Water extraction  Dosing: Bid.  Duration: 14 days  The detailed extraction procedure was not reported in manuscript. |
| 6 | Yanli Song 2013 | Shoutai pill and Shaoyao Gancao decoction | *Cuscuta chinensis* Lam. [Convolvulaceae; Cuscutae semen], 30g;  *Taxillus chinensis* (DC.) Danser [Loranthaceae; Taxilli herba], 15g;  *Dipsacus asper* Wall. ex Henry [Dipsacaceae; Dipsaci radix], 15g;  *Equus asinus* L. [Equidae; Asini corii colla], 10g;  *Paeonia lactiflora* Pall. [Ranunculaceae Juss.; Paeoniae radix alba], 20g;  *Glycyrrhiza uralensis* Fisch. (Fabaceae; Glycyrrhizae radix et rhizoma), 6g. | Administration: p.o.  Dose: 200 mL of water extraction  Dosing: Bid.  Duration: 20 days  The detailed extraction procedure was not reported in manuscript. |
| 7 | Yanwen Song 2018 | Yuetai decoction | *Rehmannia glutinosa* Libosch. [Scrophulariaceae; Rehmanniae radix], 20g;  *Scutellaria baicalensis* Georgi [Lamiaceae; Scutellariae radix], 10g;  *Phellodendron chinense* Schneid. [Cupressaceae; Phellodendri chinensis cortex], 10g;  *Paeonia lactiflora* Pall. [Ranunculaceae Juss.; Paeoniae radix alba];  *Glycyrrhiza uralensis* Fisch. [Fabaceae; Glycyrrhizae radix et rhizoma], 6g;  *Dioscorea opposita* Thunb. [Dioscoreaceae; Dioscoreae rhizoma], 12g;  *Dipsacus asper* Wall. ex Henry [Dipsacaceae; Dipsaci radix], 12g;  *Rubia cordifolia* L. [Rubiaceae; Rubiae radix et rhizoma], 10g;  *Sanguisorba officinalis* L. [Rosaceae; Sanguisorbae radix], 15g;  *Homo sapiens* [Hominidae; Crinis carbonisatus], 10g;  *SepielLa maindroni* de Rochebrune [Sepiidae; Sepiae endoconcha], 10g;  *Astragalus membranaceus* (Fisch.) Bge.var.*mongholicus* (Bge.) Hsiao [Fabaceae; Astragali radix], 10g;  *Cimicifuga heracleifolia* Kom. [Ranunculaceae; Cimicifugae rhizoma], 6g;  *Atractylodes macrocephala* Koidz. [Asteraceae; Atractylodis macrocephalae rhizoma], 10g  *Taxillus chinensis* (DC.) Danser [Loranthaceae; Taxilli herba], 10g. | Administration: p.o.  Dose: 300 mL of water extraction  Dosing: Bid.  Duration: 14 days  The detailed extraction procedure was not reported in manuscript. |
| 8 | Xin Wu 2017 | Yunbao decoction | *Dipsacus asper* Wall. ex Henry [Dipsacaceae; Dipsaci radix];  *Eucommia ulmoides* Oliv. [Eucommiaceae; Eucommiae cortex];  *Cuscuta chinensis* Lam. [Convolvulaceae; Cuscutae semen];  *Astragalus membranaceus* (Fisch.) Bge.var*.mongholicus* (Bge.) Hsiao [Fabaceae; Astragali radix];  *Atractylodes macrocephala* Koidz. [Asteraceae; Atractylodis macrocephalae rhizoma];  *Scutellaria baicalensis* Georgi [Lamiaceae; Scutellariae radix];  *Equus asinus* L. [Equidae; Asini Corii Colla];  *Paeonia lactiflora* Pall. [Ranunculaceae Juss.; Paeoniae radix alba];  *Amomum villosum* Lour. [Zingiberaceae; Amomi fructus];  *Eclipta prostrata* L. [Asteraceae; Ecliptae herba];  *Perilla frutescens* (L.) Britton [Lamiaceae; Perillae caulis];  *Sanguisorba officinalis* L. [Rosaceae; Sanguisorbae radix].  The amount of each drug in a polyherbal preparation is unavailable in original text. | Administration: p.o.  Dose: 200 mL of water extraction  Dosing: Bid.  Duration: 14 days  The detailed extraction procedure was not reported in manuscript. |
| 9 | Ping Xie 2014 | Anzi decoction | *Dipsacus asper* Wall. ex Henry [Dipsacaceae; Dipsaci radix], 15g;  *Taxillus chinensis* (DC.) Danser [Loranthaceae; Taxilli herba], 15g;  *Cuscuta chinensis* Lam. [Convolvulaceae; Cuscutae semen], 15g;  *Boehmeria nivea* (L.) Gaudich. [Urticaceae; Radix boehmeriae], 30g;  *Scutellaria baicalensis* Georgi [ Lamiaceae; Scutellariae radix], 10g;  *Atractylodes macrocephala* Koidz. [Asteraceae; Atractylodis macrocephalae rhizoma], 10g;  *Pseudostellaria heterophylla* (Miq.) Pax ex Pax et Hoffm. [Caryophyllaceae; Pseudostellariae radix], 15g;  *Glycyrrhiza uralensis* Fisch. [Fabaceae; Glycyrrhizae radix et rhizoma], 15g. | Administration: p.o.  Dose: 125 mL of water extraction  Dosing: Bid.  Duration: 10 days  The detailed extraction procedure was not reported in manuscript. |
| 10 | Jianxi Yang 2012 | Antai recipe | *Codonopsis pilosula* (Franch.) Nannf. [Campanulaceae; Codonopsis radix], 20g;  *Atractylodes macrocephala* Koidz. [Asteraceae; Atractylodis macrocephalae rhizoma], 15g;  *Paeonia lactiflora* Pall. [Ranunculaceae Juss.; Paeoniae radix alba], 15g;  Dioscorea opposita Thunb. [Dioscoreaceae; Dioscoreae rhizoma], 15g;  Citrus reticulata Blanco [Rutaceae; Citri reticulatae pericarpium], 10g;  Scutellaria baicalensis Georgi [ Lamiaceae; Scutellariae radix], 10g;  *Eucommia ulmoides* Oliv .[Eucommiaceae; Eucommiae cortex], 15g;  Rehmannia glutinosa Libosch. [Scrophulariaceae; Rehmanniae radix praeparata], 20g;  *Cuscuta chinensis* Lam. [Convolvulaceae; Cuscutae semen], 10g;  Lycium barbarum L. [Solanaceae; Lycii fructus], 10g;  Glycyrrhiza uralensis Fisch. [Fabaceae; Glycyrrhizae radix et rhizoma], 5g. | Administration: p.o.  Dose: 100 mL of water extraction  Dosing: Bid.  Duration: 10 days  The detailed extraction procedure was not reported in manuscript. |
| 11 | Limei Yu 2018 | Antai Fanglou decoction | *Cuscuta chinensis* Lam. [Convolvulaceae; Cuscutae semen], 20g;  *Codonopsis pilosula* (Franch.) Nannf. [Campanulaceae; Codonopsis radix], 15g;  Rehmannia glutinosa Libosch. [Scrophulariaceae; Rehmanniae radix praeparata], 15g;  *Taxillus chinensis* (DC.) Danser [Loranthaceae; Taxilli herba], 12g;  *Rubus chingii* Hu [Rosaceae; Rubi fructus], 10g;  *Eucommia ulmoides* Oliv. [Eucommiaceae; Eucommiae cortex], 10g;  *Atractylodes macrocephala* Koidz. [Asteraceae; Atractylodis macrocephalae rhizoma], 10g;  *Dipsacus asper* Wall. ex Henry [Dipsacaceae; Dipsaci radix], 10g;  *Paeonia lactiflora* Pall. [Ranunculaceae Juss.; Paeoniae radix alba], 6g;  *Glycyrrhiza uralensis* Fisch. [Fabaceae; Glycyrrhizae radix et rhizoma], 6g. | Administration: p.o.  Dose: Water extraction  Dosing: Bid.  Duration: 10 days  The detailed extraction procedure was not reported in manuscript. |

Abbreviations: Q.d: once per day (quaque die); Bid: twice per day (bis in die); Tid: three times per day (ter in die); p.o.: oral administration (per os.).

All taxonomical information was extracted and validated from the Chinese Pharmacopoeia 2020 version.
