# Supplementary material for: Chinese herbal medicine for threatened miscarriage: An updated systematic review and meta-analysis
Source: Front Pharmacol. 2023 Feb 14;14:1083746. doi: 10.3389/fphar.2023.1083746 (PMC9971626; doi:10.3389/fphar.2023.1083746)
Supplement: Supplementary file 1 [file DataSheet1.ZIP › Supplemental Table 1 and 2. GRADE certainty of the evidence.docx]

| **Supplemental Table 1. GRADE certainty of the evidence**   \| **CHM alone compared to WM alone for threatened miscarriage** \| \| --- \| | | | | | | | | | | | |
| --- | --- | --- | --- | --- | --- | --- | --- | --- | --- | --- | --- | --- |
| **Certainty assessment** | | | | | | | **Summary of findings** | | | | |
| **Participants (studies) Follow-up** | **Risk of bias** | **Inconsistency** | **Indirectness** | **Imprecision** | **Publication bias** | **Overall certainty of evidence** | **Study event rates (%)** | | **Relative effect (95% CI)** | **Anticipated absolute effects** | |
|  |  |  |  |  |  |  | **With WM alone** | **With CHM alone** |  | **Risk with WM alone** | **Risk difference with CHM alone** |
| **Continuation of pregnancy after 28 gestational weeks** | | | | | | | | | | | |
| 166 (1 RCT) | not serious | not serious | not serious | serious^a^ | none | ⨁⨁⨁◯ Moderate | 74/84 (88.1%) | 80/82 (97.6%) | **RR 1.11** (1.02 to 1.21) | 881 per 1,000 | **97 more per 1,000** (from 18 more to 185 more) |
| **Continuation of pregnancy after treatment** | | | | | | | | | | | |
| 922 (10 RCTs) | not serious | not serious | not serious | serious^a^ | none | ⨁⨁⨁◯ Moderate | 325/458 (71.0%) | 427/464 (92.0%) | **RR 1.30** (1.21 to 1.38) | 710 per 1,000 | **213 more per 1,000** (from 149 more to 270 more) |
| **Preterm birth** | | | | | | | | | | | |
| 0 ( studies) |  |  |  |  |  | - |  |  | not estimable | 0 per 1,000 |  |
| **Adverse maternal outcomes** | | | | | | | | | | | |
| 0 ( studies) |  |  |  |  |  | - |  |  | not estimable | 0 per 1,000 |  |
| **Adverse neonatal outcomes** | | | | | | | | | | | |
| 0 ( studies) |  |  |  |  |  | - |  |  | not estimable | 0 per 1,000 |  |
| **Neonatal death** | | | | | | | | | | | |
| 0 ( studies) |  |  |  |  |  | - |  |  | not estimable | 0 per 1,000 |  |
| **TCM syndrome severity** | | | | | | | | | | | |
| 169 (2 RCTs) | not serious | not serious | not serious | very serious^b^ | none | ⨁⨁◯◯ Low | 84 | 85 | - | - | SMD **2.94 SD lower** (4.27 lower to 1.61 lower) |
| **Improvement in β-HCG levels after treatment** | | | | | | | | | | | |
| 357 (4 RCTs) | not serious | not serious | not serious | very serious^b^ | none | ⨁⨁◯◯ Low | 178 | 179 | - | - | SMD **6.88 SD higher** (1.74 higher to 12.03 higher) |

**CI:** confidence interval; **RR:** risk ratio; **SMD:** standardized mean difference

#### Explanations

a. Downgrade one level for imprecision due to small number of participants

b. Downgraded 2 levels for imprecision due to small sample size and a wide 95% CI including benefit and minimal harm.

| **Supplemental Table 2. GRADE certainty of the evidence**  **Combined CHM-WM compared to WM alone for threatened miscarriage** | | | | | | | | | | | |
| --- | --- | --- | --- | --- | --- | --- | --- | --- | --- | --- | --- |
| **Certainty assessment** | | | | | | | **Summary of findings** | | | | |
| **Participants (studies) Follow-up** | **Risk of bias** | **Inconsistency** | **Indirectness** | **Imprecision** | **Publication bias** | **Overall certainty of evidence** | **Study event rates (%)** | | **Relative effect (95% CI)** | **Anticipated absolute effects** | |
|  |  |  |  |  |  |  | **With WM alone** | **With combined CHM-WM** |  | **Risk with WM alone** | **Risk difference with combined CHM-WM** |
| **Continuation of pregnancy after 28 weeks of gestation** | | | | | | | | | | | |
| 1519 (15 RCTs) | not serious | not serious | not serious | serious^a^ | none | ⨁⨁⨁◯ Moderate | 579/759 (76.3%) | 702/760 (92.4%) | **RR 1.21** (1.16 to 1.27) | 763 per 1,000 | **160 more per 1,000** (from 122 more to 206 more) |
| **Continuation of pregnancy after treatment** | | | | | | | | | | | |
| 4372 (41 RCTs) | not serious | not serious | not serious | serious^a^ | none | ⨁⨁⨁◯ Moderate | 1625/2107 (  77.1%) | 2086/2265 (92.1%) | **RR 1.19** (1.16 to 1.23) | 771 per 1,000 | **147 more per 1,000** (from 123 more to 177 more) |
| **Preterm birth** | | | | | | | | | | | |
| 1031 (9 RCTs) | not serious | not serious | not serious | very serious^b^ | none | ⨁⨁◯◯ Low | 101/515 (19.6%) | 50/516 (9.7%) | **RR 0.50** (0.36 to 0.68) | 196 per 1,000 | **98 fewer per 1,000** (from 126 fewer to 63 fewer) |
| **Adverse maternal outcomes** | | | | | | | | | | | |
| 707 (8 RCTs) | not serious | not serious | not serious | very serious^b^ | none | ⨁⨁◯◯ Low | 33/353 (9.3%) | 32/354 (9.0%) | **RR 0.97** (0.62 to 1.52) | 93 per 1,000 | **3 fewer per 1,000** (from 36 fewer to 49 more) |
| **Adverse neonatal outcomes** | | | | | | | | | | | |
| 0 ( studies) |  |  |  |  |  | - |  |  | not estimable | 0 per 1,000 |  |
| **Neonatal death** | | | | | | | | | | | |
| 246 (2 RCTs) | not serious | not serious | not serious | very serious^b^ | none | ⨁⨁◯◯ Low | 10/119 (8.4%) | 4/127 (3.1%) | **RR 0.39** (0.12 to 1.21) | 84 per 1,000 | **51 fewer per 1,000** (from 74 fewer to 18 more) |
| **TCM syndrome severity** | | | | | | | | | | | |
| 1475 (16 RCTs) | not serious | not serious | not serious | very serious^b^ | none | ⨁⨁◯◯ Low | 736 | 739 | - | - | SMD **2.86 SD lower** (3.48 lower to 2.23 lower) |
| **Improvement in β-HCG levels after treatment** | | | | | | | | | | | |
| 3957 (37 RCTs) | not serious | not serious | not serious | very serious^b^ | none | ⨁⨁◯◯ Low | 1927 | 2030 | - | - | SMD **2.27 SD higher** (1.72 higher to 2.83 higher) |

**CI:** confidence interval; **RR:** risk ratio; **SMD:** standardized mean difference

#### Explanations

a. Downgrade one level for imprecision due to small sample size.

b. Downgraded two levels for imprecision due to small sample size and a wide 95% CI including benefit and minimal harm.
