# Supplementary material for: Chinese herbal medicine for threatened miscarriage: An updated systematic review and meta-analysis
Source: Front Pharmacol. 2023 Feb 14;14:1083746. doi: 10.3389/fphar.2023.1083746 (PMC9971626; doi:10.3389/fphar.2023.1083746)
Supplement: Supplementary file 1 [file DataSheet1.ZIP › Supplemental Table 3-Summary of adverse events.docx]

**Supplemental Table 3**

| **Study ID** | **Intervention** | **Sample size** | **Adverse events** | | | | | | | **Serious adverse events** | | **Adverse events rate** |
| --- | --- | --- | --- | --- | --- | --- | --- | --- | --- | --- | --- | --- |
|  |  |  | Mild diarrhea | Rash | Dizziness/  headache | Nausea/  vomiting | Constipation | Mouth dryness | Poor appetite | Insomnia | Painful breasts |  |
| Dong 2021 | CHM-WM | 50 | NR | 1 | 1 | 2 | NR | NR | NR | 2 | 1 | 7/50 |
| Dong 2021 | WM | 50 | NR | 2 | 0 | 1 | NR | NR | NR | 1 | 2 | 6/50 |
| Huang 2020 | CHM-WM | 39 | 0 | NR | NR | 2 | NR | NR | NR | NR | NR | 2/39 |
| Huang 2020 | WM | 39 | 1 | NR | NR | 0 | NR | NR | NR | NR | NR | 1/39 |
| Liu b 2020 | CHM-WM | 30 | NR | 0 | 1 | 2 | NR | NR | NR | NR | NR | 3/30 |
| Liu b 2020 | WM | 30 | NR | 2 | 3 | 4 | NR | NR | NR | NR | NR | 9/30 |
| Ma 2019 | CHM-WM | 64 | NR | NR | 2 | 3 | 1 | NR | NR | NR | NR | 6/64 |
| Ma 2019 | WM | 64 | NR | NR | 2 | 2 | 0 | NR | NR | NR | NR | 4/64 |
| Shang 2021 | CHM-WM | 40 | NR | NR | 1 | 2 | 1 | 1 | NR | NR | NR | 5/40 |
| Shang 2021 | WM | 40 | NR | NR | 2 | 1 | 2 | 1 | NR | NR | NR | 6/40 |
| Wang 2019 | CHM-WM | 40 | NR | NR | NR | 2 | NR | NR | NR | NR | NR | 2/40 |
| Wang 2019 | WM | 40 | NR | NR | NR | 0 | NR | NR | NR | NR | NR | 0/40 |
| Zhang 2020b | CHM-WM | 42 | NR | NR | NR | 2 | 1 | 3 | 0 | NR | NR | 6/42 |
| Zhang 2020b | WM | 42 | NR | NR | NR | 3 | 1 | 2 | 1 | NR | NR | 7/42 |
| Zhu 2019 | CHM-WM | 51 | NR | NR | NR | 1 | NR | NR | NR | NR | NR | 1/51 |
| Zhu 2019 | WM | 51 | NR | NR | NR | NR | NR | NR | NR | NR | NR | 0/51 |

**Summary of adverse events in the included studies**

NR: not reported
