# Supplementary figures and images for: Chinese herbal medicine for threatened miscarriage: An updated systematic review and meta-analysis
Source: Front Pharmacol. 2023 Feb 14;14:1083746. doi: 10.3389/fphar.2023.1083746 (PMC9971626; doi:10.3389/fphar.2023.1083746)

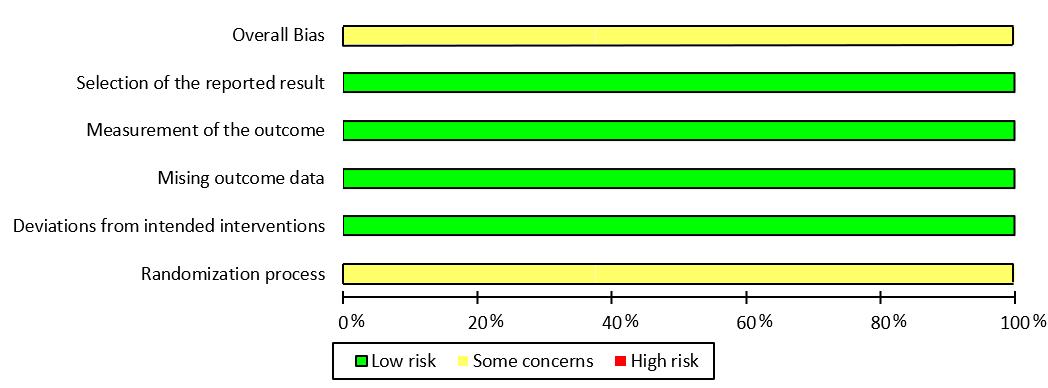

Supplement: Supplementary file 1 [file DataSheet1.ZIP › Supplemental Figure 1. Risk of bias graph.jpg]

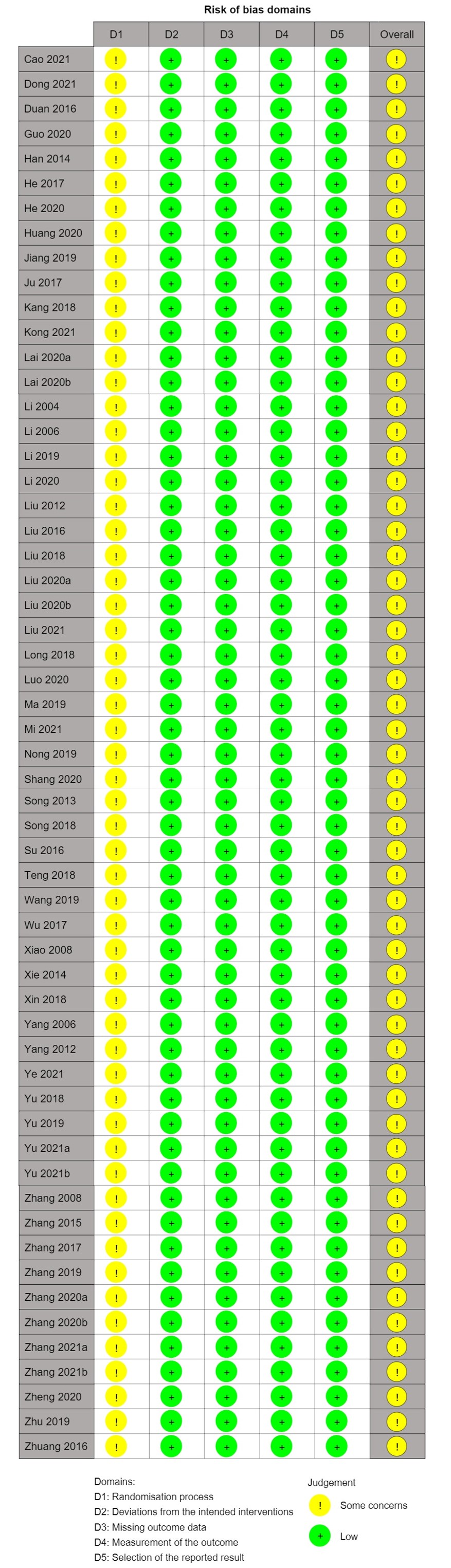

Supplement: Supplementary file 1 [file DataSheet1.ZIP › Supplemental Figure 2. Risk of bias summary.jpg]
