# Supplementary material for: Chinese herbal medicine for threatened miscarriage: An updated systematic review and meta-analysis
Source: Front Pharmacol. 2023 Feb 14;14:1083746. doi: 10.3389/fphar.2023.1083746 (PMC9971626; doi:10.3389/fphar.2023.1083746)
Supplement: Supplementary file 1 [file DataSheet1.ZIP › Cover letter.docx]

**Corresponding author**: Professor Lu Li

**Affiliation (Postal Address**): Pharmaceutical Informatics Institute, College of Pharmaceutical Sciences, Zhejiang University, Hangzhou, 310058, China.

**Tel**: 0571-88208596.

**E-mail**: luciali@zju.edu.cn.

Hangzhou, Zhejiang, China. 14/10/2022

Dear Frontiers in Pharmacology editors,

On behalf of all authors, I am delighted to submit a manuscript entitled ‘Chinese herbal medicine for threatened miscarriage: an updated systematic review and meta-analysis’ to your appreciation and to the editorial analysis of the Frontiers in Pharmacology, as to the possibility of its publication.

This manuscript is based on no current medicines that can effectively prevent threatened miscarriage. Among these, Chinese herbal medicine has shown its therapeutical effects. However, its efficacy and safety remain elusive due to the poor quality of previous trials. This study aimed to perform an updated systematic review and meta-analysis on the efficacy and safety of CHM for threatened miscarriage.

In this paper, 57 randomized clinical trials involving 5881 patients with threatened miscarriage were included for meta-analysis, which indicated a significant improvement in the efficacy and no increase in the adverse events of combined Chinese Herbal Medicine and Western Medicine therapy for threatened miscarriage compared with Western Medicine alone. We believe this manuscript is valuable for all the researchers who are interested.

This manuscript has not been published before and is not being considered for publication elsewhere, all authors have contributed to the creation of this manuscript for important intellectual content and read and approved the final manuscript. We declare is no conflict of interest.

Thank you for receiving our manuscript and considering it for review. We deeply appreciate your time and look forward to your response.

Yours Sincerely,
